# Age‐related loss of intestinal barrier integrity plays an integral role in thymic involution and T cell ageing

**DOI:** 10.1111/acel.14401

**Published:** 2024-11-15

**Authors:** Jessica Conway, Erica N. De Jong, Andrea J. White, Ben Dugan, Nia Paddison Rees, Sonia M. Parnell, Lisa E. Lamberte, Archana Sharma‐Oates, Jack Sullivan, Claudio Mauro, Willem van Schaik, Graham Anderson, Dawn M. E. Bowdish, Niharika A. Duggal

**Affiliations:** ^1^ Institute of Inflammation and Ageing University of Birmingham Birmingham UK; ^2^ Department of Medicine McMaster University Hamilton Ontario Canada; ^3^ Institute for Immunology and Immunotherapy University of Birmingham Birmingham UK; ^4^ Institute of Microbiology and Infection University of Birmingham UK; ^5^ School of Biosciences University of Birmingham Birmingham UK

**Keywords:** immunesenescence, intestinal barrier leakage, T cell ageing, thymic involution

## Abstract

The intestinal epithelium serves as a physical and functional barrier against harmful substances, preventing their entry into the circulation and subsequent induction of a systemic immune response. Gut barrier dysfunction has recently emerged as a feature of ageing linked to declining health, and increased intestinal membrane permeability has been shown to promote heightened systemic inflammation in aged hosts. Concurrent with age‐related changes in the gut microbiome, the thymic microenvironment undergoes a series of morphological, phenotypical and architectural alterations with age, including disorganisation of the corticomedullary junction, increased fibrosis, increased thymic adiposity and the accumulation of senescent cells. However, a direct link between gut barrier dysbiosis and thymic involution leading to features of immune ageing has not been explored thus far. Herein, we reveal strong associations between enhanced microbial translocation and the peripheral accumulation of terminally differentiated, senescent and exhausted T cells and the compensatory expansion of regulatory T cells in older adults. Crucially, we demonstrate that aged germ‐free mice are protected from age‐related increases in intestinal permeability, highlighting the direct impact of mucosal permeability on thymic ageing. Together, these findings establish a novel mechanism by which gut barrier dysfunction drives systemic activation of the immune system during ageing through thymic involution. This enhances our understanding of drivers of T cell ageing and opens up the possibility for the use of microbiome‐based interventions to restore immune homeostasis and promote healthy ageing in older adults.

## Graphical abstract

Loss of intestinal barrier integrity with age permits the translocation of microbes and microbial products, such as LPS, into the circulation. These microbial products are then trafficked through the blood, contributing to persistent antigenic stimulation that leads to the accumulation of senescent T cells. Through their senescence‐associated secretory phenotype (SASP), senescent T cells promote systemic inflammation and induce a bystander effect in the thymus, contributing to age‐related thymic involution and a reduced thymic output of naïve T cells (RTEs). Loss of naïve T cells and chronic antigenic stimulation result in skewing towards T cell senescence, which accelerates immunological ageing (high IMM‐AGE score) in older adults.

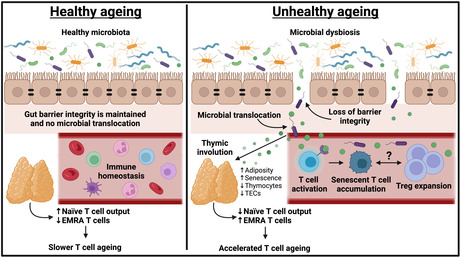

**Abbreviations**
ANOVA: analysis of variance
BMI: Body Mass Index
CMV: Cytomegalovirus
CRP: C reactive protein
DCA: Deoxycholic acid
EMRA: effector memory re‐expressing RA
GDCA: glycodeoxycholic acid
HADS: Hospital Anxiety and Depression Score; I
i‐ FABP: intestinal fatty‐acid binding protein
IgG: immunoglobulin G
LBP: lipopolysaccharide‐binding protein
LCA: lithocholic acid
MET: metabolic equivalent of task
MT: microbial translocation
OTU: operational taxonomic units
PBMC: peripheral blood mononuclear cells
PSQI: Pittsburgh Sleep Quality Index.
RNA: Ribonucleic acid
RTE: recent thymic emigrants
SCFA: Short‐chain fatty acids
TEC: thymic epithelial cells
TNFα: tumour necrosis factor alpha
UDCA: Ursodeoxycholic acid

## 1 BACKGROUND

The epithelium of the gastrointestinal tract represents the largest mucosal lining in the body that effectively limits the permeation of luminal microorganisms, antigens and toxins through its paracellular space, a process that is regulated by intercellular tight junctions. Advancing age is accompanied by physiological changes to the intestine, including mucus layer thinning and remodelling of intestinal epithelial tight junction proteins, such as zonulin, which contribute towards the breakdown of intestinal barrier function in aged worms, flies, fish, rodents, non‐human primates and humans; permitting commensal bacteria and their products, such as LPS, from the gut lumen into the bloodstream (referred to as a leaky gut) (Salazar et al., 2023). Age‐related intestinal barrier dysfunction is closely linked to the progressive deterioration of systemic health and the gradual appearance of metabolic defects (Wilson et al., 2018). Moreover, recent evidence from animal studies indicates that it is a major contributor to low‐grade systemic inflammation, termed inflammaging (Thevaranjan et al., 2017). Human intestinal barrier dysfunction, determined by elevated circulating lipopolysaccharide‐binding protein (LBP) levels, is also associated with impaired physical function and inflammaging in healthy aged adults (Stehle Jr. et al., 2012); highlighting the importance of investigating the role of intestinal barrier dysfunction in ageing.

Concurrently with changes to intestinal homeostasis, ageing is accompanied by remodelling of the immune system that attenuates the host's ability to mount robust immune responses, resulting in an immunocompromised state, termed immunesenescence. Age‐related immune dysfunction contributes towards increased susceptibility to poor outcomes during bacterial and viral infections, elevated risk of autoimmunity, poor response to vaccination; increasing the risk of morbidity and mortality in older adults (Duggal, 2018). One of the most striking features of immune ageing is the progressive shrinkage (involution) of the thymus that is characterised by the loss of thymic epithelial cells (TECs), expansion of perivascular spaces, increased thymic adiposity and the accumulation of senescent cells; together resulting in a loss of functional spaces for the development of thymocytes (Aw et al., 2008). In addition to thymic architectural disorganisation, alterations in the thymic stromal cell microenvironment, including elevated levels of thymopoiesis‐suppressing cytokines (e.g. IL6 and tumour necrosis factor alpha (TNFα) also occur with age (Palmer, 2013). Collectively this compromises the process of thymopoiesis and result in a reduced thymic output of naïve T cells and the homeostatic expansion of peripheral memory T cell subsets (Saule et al., 2006). Further, chronic lifelong antigenic stimulation leads to the accumulation of senescent T cells in the periphery (Pangrazzi et al., 2020), which impair tissue immunosurveillance and drive a state of prolonged basal inflammation in aged individuals, termed inflammageing. This is further exacerbated by the age‐related expansion of pro‐inflammatory Th17 cells (Lim et al., 2014) and anti‐inflammatory regulatory T cells (Tregs) (Elyahu et al., 2019).

Despite these interesting findings, the relationship between intestinal barrier dysfunction and immune ageing is poorly understood. Herein we report that intestinal membrane permeability increases with age in humans and is accompanied by enhanced systemic microbial translocation that contributes to the lifelong antigenic burden, driving a reduction in naïve T cell thymic output and an accumulation of terminally differentiated, senescent T cells in the periphery. The emergence of these hallmarks of T cell ageing hinders the ability of these cells to fight invading pathogens and enhances their ability to produce pro‐inflammatory cytokines, which ultimately contribute to the inflammatory state of the aged host. Further, we demonstrate that aged germ‐free mice, which do not exhibit age‐related intestinal barrier dysfunction, are protected from the accumulation of microbial products in the thymus and maintain their thymic architecture. Together, these findings provide novel evidence of a causal relationship between intestinal barrier dysfunction and T cell ageing.

## 2 RESULTS

### 2.1 Ageing is accompanied by increased microbial translocation

**FIGURE 1:**
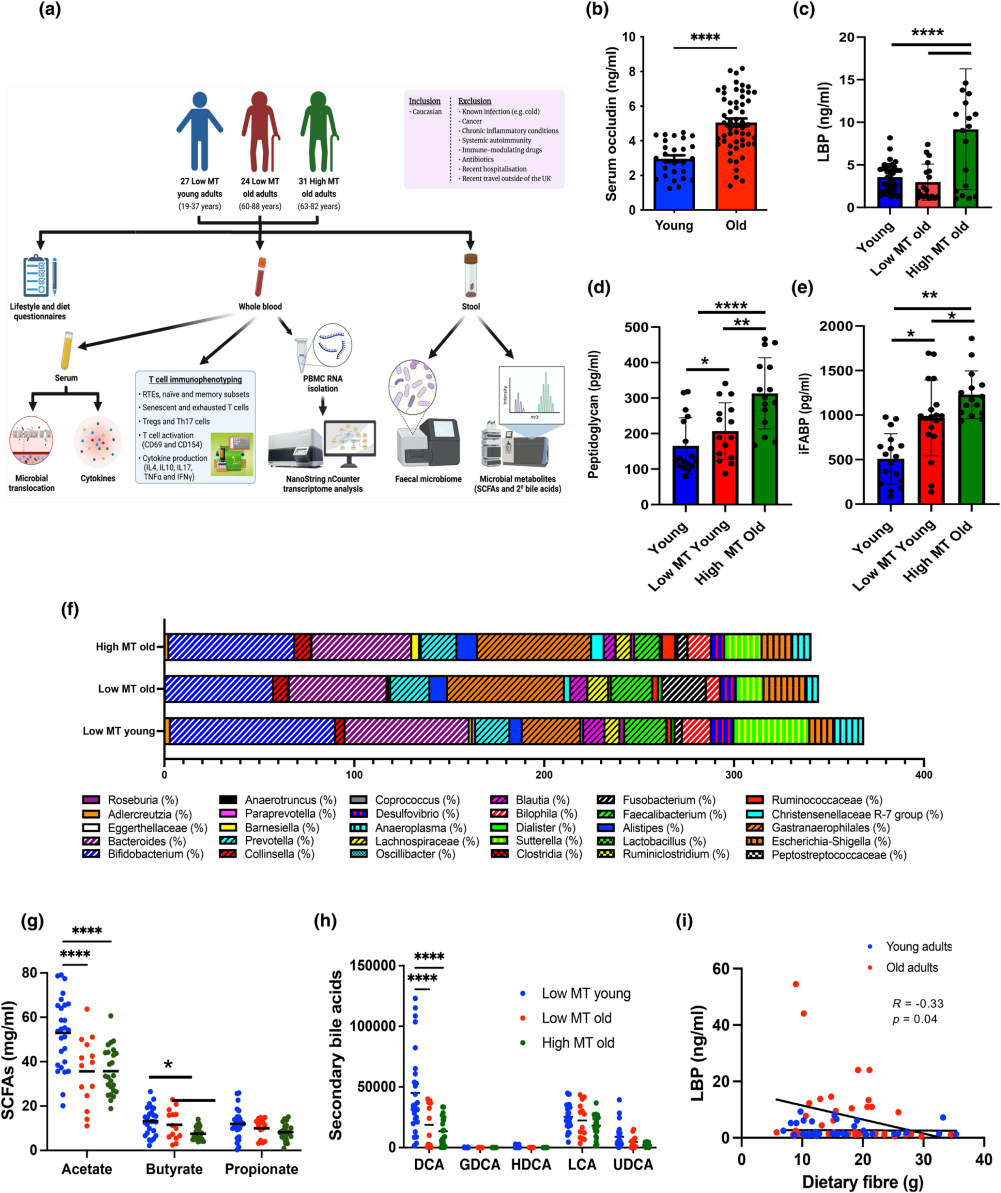
Age‐related microbial translocation and links with gut microbiome composition and dietary components (a) Study design and exclusion criteria for participant recruitment. Circulating levels of occludin (b) in young (*n* = 27) and old (*n* = 55) participants. Unpaired Student's T test and Mann‐Whitney *U* test were used. (c) LBP (d) Peptidoglycan (e) i FABP in low MT young (blue), low MT old (red) and high MT old (green) adults. (f) Faecal relative abundance of bacterial species in the genus (%) (g) short‐chain fatty acids (SCFAs) (h) and secondary bile acids in low MT young (blue), low MT old (red) and high MT old (green) adults. (i) Spearman‐based correlations between LBP levels and dietary intake. One‐way ANOVA with Bonferroni's multiple comparison test was used. *p ≤ 0.05, **p < 0.01, ****p ≤ 0.0001. DCA, deoxycholic acid; HDCA, hyodeoxycholic acid; LCA, lithocholic acid; UDCA, ursodeoxycholic acid.

Twenty‐seven healthy young individuals (age range 19–37 years) and 55 community‐dwelling healthy old individuals (age range 63–84 years) were recruited into this study, from whom blood samples were collected to assess microbial translocation and immune cell profiles (Figure 1a). Occludin is an integral tight junction protein located on the basolateral membrane of intestinal epithelial cells, whose presence in the circulation is a biomarker of increased intestinal membrane permeability (Groschwitz & Hogan, 2009). In this study, we found a significant age‐associated increase in circulating occludin levels (*p* < 0.0001) (Figure 1b). Serum LBP is a surrogate biomarker of intestinal permeability and microbial translocation (Stehle Jr. et al., 2012). We observed a trend towards elevated circulating LBP levels with age; however, this did not reach statistical significance (*p* = 0.12) (Figure 1c). Interestingly, elevated circulating LBP levels were linked to low dietary fibre intake (*R* = −0.33, *p* = 0.04) (Figure 1d).

We next investigated potential associations between markers of microbial translocation and potential drivers of increased gut permeability, including age, sex, body mass index (BMI), diet, physical activity levels, sedentary behaviour, sleep quality and mental health (Table S1). Upon assessment of the faecal microbiome, older adults displaying high microbial translocation had lower relative abundances of *Bifidobacterium* (*p* = 0.0002), *Blautia* (*p* = 0.002) and *Lachnospiraceae* (*p* = 0.01) and greater relative abundances of *Christensenellaceae R‐7 group* (*p* = 0.02) and *Ruminococcaceae* (*p* = 0.03) in stool compared to young adults (Figure 1e; Table S2). Low levels of the short‐chain fatty acid butyrate (*p* = 0.02), propionate (*p* = 0.03) and the secondary bile acid glycodeoxycholic acid (GDCA) (*R* = −0.33, *p* = 0.04) in stool were also strongly linked with elevated circulating levels of occludin in older adults (Figure 1f,g, respectively).

Furthermore, were split our older adults' cohort into two sub‐groups: low Microbial Translocation (MT) old (*n* = 24, age range 60–88 years) and high MT old (*n* = 31, age range 63–82 years) (Figure 1a). Low MT (similar levels to healthy young individuals) was defined as circulating occludin levels ≤4.5 ng/mL, which was the mean value observed in young participants. High MT was defined as circulating occludin levels >4.5 ng/mL. Demographic characteristics for each subgroup are shown in Table 1.

**TABLE 1:** Participant demographics.

	Low MT young (*n* = 27)	Low MT old (*n* = 24)	High MT old (*n* = 31)	*p*‐value
Age (years)	25.8 ± 1	70.6 ± 1.6	73 ± 1	**<0.0001**
Male, *n* (%)	17 (37%)	11 (54%)	9 (29%)	**0.03**
BMI (kg/m^2^)	26.5 ± 1.3	25.4 ± 1.4	24.4 ± 0.8	0.22
Smokers, *n* (%)	1 (4%)	0 (0%)	0 (0%)	0.36
Alcohol consumption (units per drink per week)	7.5 ± 1.6	8.2 ± 2.2	4.1 ± 1.2	0.12
Physical activity levels (MET)	50 ± 5.6	33.3 ± 6.9	35 ± 4	0.07
Sedentary TV viewing time (h)	20.9 ± 1.8	17.4 ± 3.1	17.7 ± 2	0.23
PSQI score	4.6 ± 2.6	4.3 ± 0.5	5.1 ± 2.9	0.78
HADS anxiety score	6.2 ± 0.8	3.4 ± 0.5	2.9 ± 0.4	**0.001**
HADS depression score	3 ± 0.6	1.6 ± 0.4	2.2 ± 0.5	0.49
Mediterranean Diet Score	5 ± 0.5	7.1 ± 0.6	6.6 ± 0.5	**0.01**
Diet Quality Index	62 ± 1.5	66.8 ± 1.5	64.6 ± 1.6	0.12
CMV IgG (antibody index)	0.8 ± 0.2	0.8 ± 3	0.8 ± 0.2	0.79
CMV IgG seropositivity, *n* (%)	10 (37%)	5 (21%)	10 (32%)	0.44

*Note*: Data are mean ± standard error mean. One‐way ANOVA with Bonferroni's multiple comparison test and Dunn's multiple comparison test along with Chi‐square test were used. Significant *p*‐values are highlighted in red.

Abbreviations: BMI, body mass index; CMV, cytomegalovirus; HADS, Hospital Anxiety and Depression Score; IgG, immunoglobulin G; MET, metabolic equivalent of task; PSQI, Pittsburgh Sleep Quality Index.

### 2.2 T cell immunesenescence and microbial translocation

In this study, we hypothesised that an increase in circulating microbial products would perpetuate repeated T cell activation and, the subsequent differentiation of T cells and induction of replicative senescence, all recognised as features of T cell ageing. Therefore, we reasoned that older adults with lower levels of intestinal barrier leakage would exhibit fewer features of T cell ageing.

**FIGURE 2:**
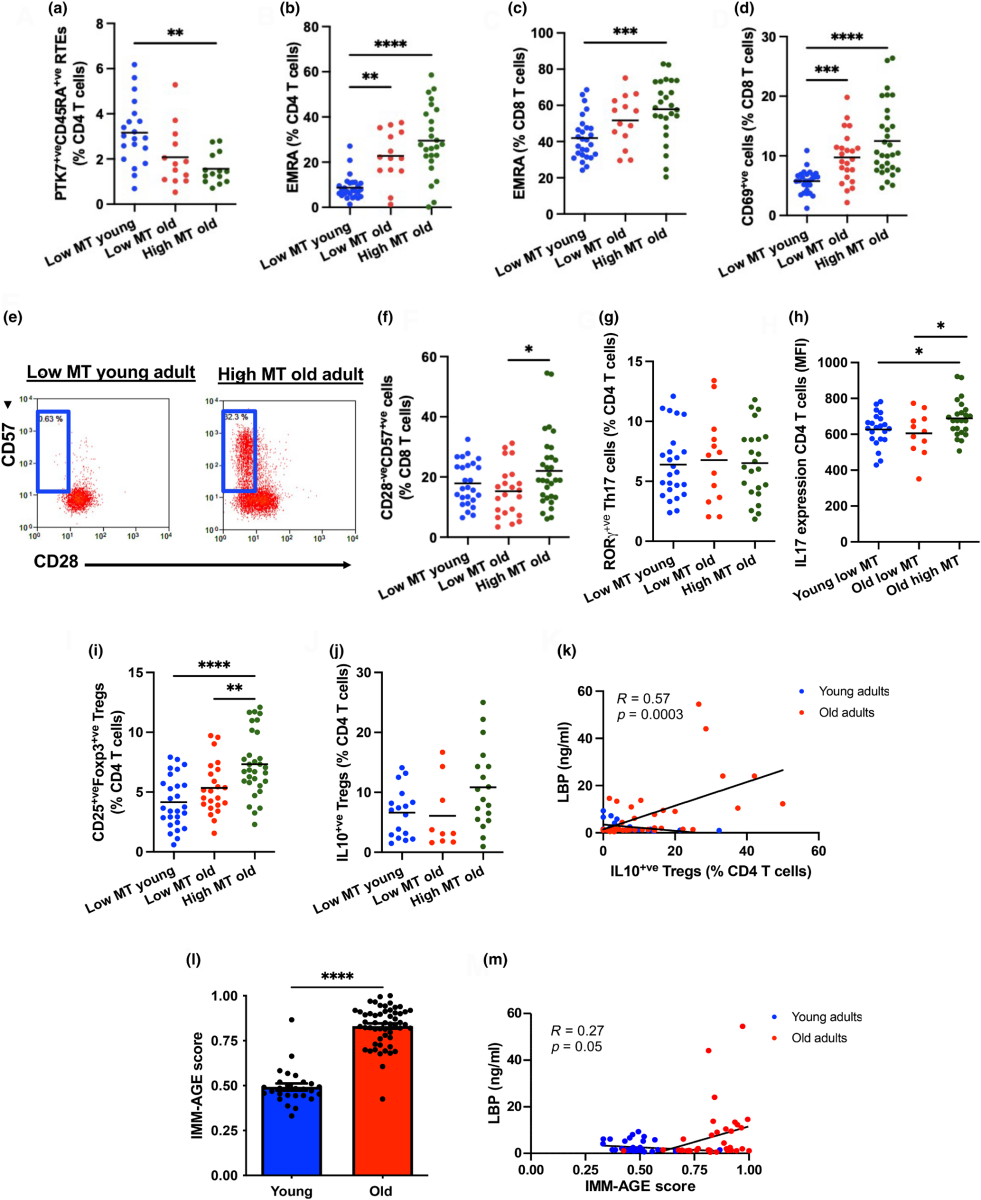
Age‐related microbial translocation and T cell subset distribution. (a) The percentage of PTK7^+ve^CD45RA^+ve^ RTEs within the CD4 T cell pool in low MT young adults (*n* = 20), low MT old adults (*n* = 14) and high MT old adults (*n* = 15). (b) The proportion of EMRA CD4 T cells and CD8 T cells (c) and CD69^+ve^ CD8 T cells (d). (e) Representative flow cytometric plots showing CD28^−ve^CD57^+ve^ senescent CD8 T cells in a young adult with low microbial translocation and a healthy old individual displaying high microbial translocation. (f) The peripheral frequency of CD28^−ve^CD57^+ve^ senescent CD8 T cells. (g) The percentage of RORγ^+ve^ Th17 cells in low MT young adults (*n* = 25), low MT old adults (*n* = 13) and high MT old adults (*n* = 23). (h) Intracellular IL17 expression levels (MFI) in CD4 T cells from young (*n* = 22) and old (*n* = 11) donors with low MT and high MT (*n* = 24). (i) The peripheral frequency of CD25^+ve^Foxp3^+ve^ Tregs. (j) Intracellular IL10 expression levels (MFI) in CD4 T cells from young (*n* = 22) and old (*n* = 11) donors with low MT and high MT (*n* = 24). Means are shown as solid lines. One‐way ANOVA with Bonferroni's multiple comparison test and Dunn's multiple comparisons test were used. *p ≤ 0.05, **p ≤ 0.01, ***p ≤ 0.001, ****p ≤ 0.0001. (k) Linear regression plot showing correlations (Spearman) between the circulating LBP levels and the and IL10‐producing Tregs. (l) Comparison of immunological age (IMM‐AGE score) between young (*n* = 40) and old (*n* = 40) volunteers using Unpaired Student's *t* test. ****p ≤ 0.0001. (m) Spearman's rank‐based correlation plots depicting associations between surrogate markers for intestinal barrier dysfunction and the IMM‐AGE score.

Previous studies have reported an age‐related decline in peripheral PTK7^+ve^CD45RA^+ve^ recent thymic emigrants (RTEs), which are antigenically naïve CD4 T cells that egress from the thymus into the periphery following intrathymic development and thus a surrogate marker for human thymic output (Haines et al., 2009). Interestingly, we report that there is a significant reduction in the proportion of circulating RTEs in the presence of high microbial translocation in older adults (*p* = 0.002) compared to young participants, but not in those with low MT (Figure 2a). Further, older participants with high MT presented with greater proportions of central memory CD8 T cells (*p* = 0.03), effector memory CD4 and CD8 T cells (*p* < 0.0001 for both) (Tables S3 and S4) and terminally differentiated effector memory re‐expressing RA (EMRA) CD4 (*p* < 0.0001) and CD8 T cells (*p* = 0.0005; Figure 2b,c), compared to low MT young individuals.

CD69 is an early activation marker expressed by activated lymphocytes. Here, we observed an age‐associated increase in the percentage of CD69^+ve^ CD8 T cells (*p* = 0.01), which was even greater in older adults displaying high MT (*p* < 0.0001) (Figure 2d); suggesting that translocated bacterial might induce polyclonal T cell activation.

Advancing age is accompanied by the loss of CD28 and the gain of CD57 expression on the surface of CD8 T cells (known markers of T cell senescence), which have low proliferative capacity and are highly pro‐inflammatory (Pangrazzi et al., 2020). In this study, we observed a significant increase in CD28^−ve^CD57^+ve^ senescent CD8 T cells in older adults with high MT compared to old adults with low MT (*p* = 0.04) (Figure 2e,f). A similar increase in CD28^−ve^CD57^+ve^ senescent CD4 T cells was also seen in older adults displaying high MT relative to young adults (*p* = 0.02) (Table S3). Another hallmark of T cell immunesenescence is an increase in programmed cell death‐1 (PD1)^+ve^ exhausted CD8 T cells with reduced cytotoxic capability and reduced proliferative potential (Lee et al., 2016). Older adults with low MT possessed significantly greater percentages of PD1^+ve^ CD8 T cells compared to young adults (*p* = 0.04), but not older donors with high MT (*p* = 0.6) (Table S4).

**FIGURE 3:**
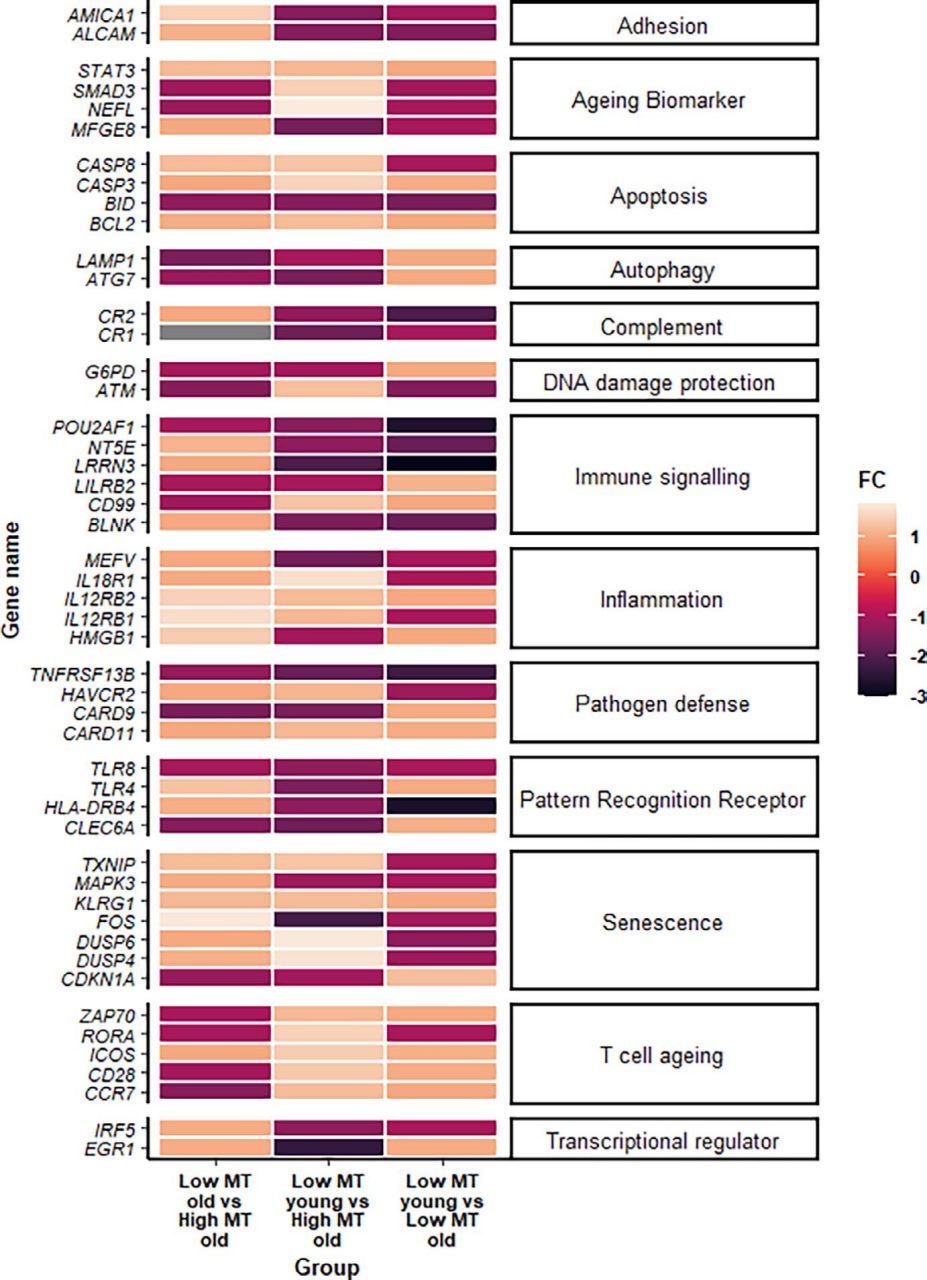
Molecular mechanisms underpinning microbial translocation‐induced T cell ageing. Heat map showing fold change log(2) of differentially expressed genes in low MT young (*n* = 6), low MT old (*n* = 6) and high MT old (*n* = 6) participants.

CD4 T helper cells are important mediators of inflammatory responses, secreting effector cytokines upon activation. RORγ^+ve^ Th17 cells, defined by their ability to secrete IL17, are pro‐inflammatory and have been associated with several autoimmune disorders (Zizzo et al., 2011). Upon examination of CD4 T helper cell subsets, we observed comparable proportions of RORγ^+ve^ Th17 cells (*p* = 0.94) (Figure 2g) between the three participant groups, but older adults with increased microbial translocation had higher intracellular IL17 expression levels than young and old participants displaying low MT (*p* = 0.04 for both) (Figure 3h; gating strategy Figure S1).

CD25^+ve^Foxp3^+ve^ Tregs are another subpopulation of CD4 T helper cells that maintain homeostasis and immune tolerance through multiple mechanisms, one of them being secretion of the anti‐inflammatory cytokine IL10 (Wei et al., 2017). In this study, the peripheral frequency of CD25^+ve^Foxp3^+ve^ Tregs was significantly higher in high MT older adults compared to young and old participants with low MT (*p* < 0.0001 and *p* = 0.01, respectively) (Figure 2i). However, the proportion of anti‐inflammatory IL10‐producing Tregs (*p* = 0.21) and intracellular IL10 expression levels (*p* = 0.6) were similar between the three groups (Figure 2j; Table S3). Treg expansion is thought to occur as a compensatory mechanism against a pro‐inflammatory cytokine milieu, as is the case with advancing age. Further, peripheral LBP levels were positively associated with the accumulation of IL10‐expressing Tregs (*R* = 0.57, *p* = 0.0003) (Figure 2k) in older people.

On assessing the impact of microbial translocation on serum cytokine levels, circulating concentrations of IL1β, IL4, IL6, IL15, TNFα, CRP, IFNγ, CXCL9 and GM‐CSF were unaltered by advancing age and the presence of intestinal barrier dysfunction (Table S5). Although the peripheral frequency of Th17 cells and intracellular IL17 levels were comparable between groups, older adults with high MT displayed lower systemic levels of IL17 compared to other participants with low MT (*p* = 0.02) (Table S5).

### 2.3 Links between microbial translocation and the IMM‐AGE score

The IMM‐AGE score is a recently developed metric that describes an individual's cellular immune profile about their chronological age and has been recognised as a reliable predictor of all‐cause mortality in older adults (Alpert et al., 2019). Here, we used a modified version that requires only eight T cell subsets: total T cells, naïve CD4 T cells, effector memory CD4 and CD8 T cells, EMRA CD8 T cells, CD28^−ve^ CD8 T cells, CD57^+ve^ CD8 T cells and Tregs (Foster et al., 2022). IMM‐AGE scores were significantly higher in older adults relative to young controls (*p* < 0.0001) (Figure 2l). When investigating potential associations between IMM‐AGE scores and markers of gut permeability and microbial translocation, we found that high IMM‐AGE scores were positively correlated with circulating LBP (*R* = 0.28, *p* = 0.05) (Figure 2m).

### 2.4 Transcriptome signatures of older adults with low and high microbial translocation

To identify molecular signalling pathways in peripheral immune cells that might contribute towards enhanced immune ageing in older adults with high microbial translocation, we used the NanoString nCounter® gene expression assay. This allowed for the detection of 770 genes in peripheral blood mononuclear cells (PBMCs) from healthy young individuals displaying low MT (*n* = 6), healthy old individuals with low MT (*n* = 6) and healthy old individuals displaying high MT (*n* = 6).

In total, 49 genes significantly differed between low MT young, low MT old and high MT old participants (Figure 3). Upon comparing expression levels between low MT young adults and older adults with low MT, we observed a significant downregulation in the expression of seven genes controlling cell adhesion/migration (ALCAM, *p* = 0.05), apoptosis (BID, *p* = 0.04), immune suppression (LRRN3, *p* = 0.05) and immune‐mediated pathology (NT5E, *p* = 0.04; TNFRSF13B, *p* = 0.05) (Table S6).

Furthermore, 27 genes were differentially expressed between young individuals and high MT older adults (Table S7). For instance, we observed downregulated expression of autophagy‐related genes (ATG7, *p* = 0.05; LAMP, *p* = 0.03) and DNA repair machinery (ATM, *p* = 0.05; G6PD, *p* = 0.03) along with an upregulation of pro‐apoptotic molecules (CASP3, *p* = 0.05; BID, *p* = 0.04) only in aged individuals with high MT (Figure 3). Importantly, we saw increased expression of co‐stimulatory molecules expressed on activated T cells (ICOS, *p* = 0.05), cellular senescence markers (gain of KLRG1, *p* = 0.04; loss of CD28, *p* = 0.05) and accelerators of cell cycle arrest (TXNIP, *p* = 0.05) only in older adults with high MT (Figure 3). It was previously reported that p53 inhibits mitogen‐activated protein kinase (MAPK) activity by inducing phosphatases, such as the dual‐specificity phosphatases (DUSPs) (Ueda et al., 2003). Supporting this, we observed a downregulation of MAPK3 (*p* = 0.04) and upregulated expression of DUSP4 (*p* = 0.04) and DUSP6 (*p* = 0.05) in older adults with high MT relative to young and aged individuals with low MT (Table S8). Additionally, older participants with high MT displayed increased expression of inflammatory molecules (HMBG1, *p* = 0.01) and T cell ageing markers (RORA, *p* = 0.03) compared to low MT older adults (Figure 3).

Gene enrichment analysis revealed that the top enriched terms in high MT olds were the intrinsic pathways for apoptosis (BCL2, BID and CD99), the cytochrome c‐mediated apoptotic response (CASP3 and CASP8), cellular senescence (CD28, DUSP4, DUSP6, KLRG1, MAPK3 and TXNIP), suggesting that these pathways might be involved in driving T cell immunesenescence during age‐related intestinal barrier dysfunction.

### 2.5 Thymic tissue architecture, adiposity and senescence in aged germ‐free mice protected from intestinal permeability

**FIGURE 4:**
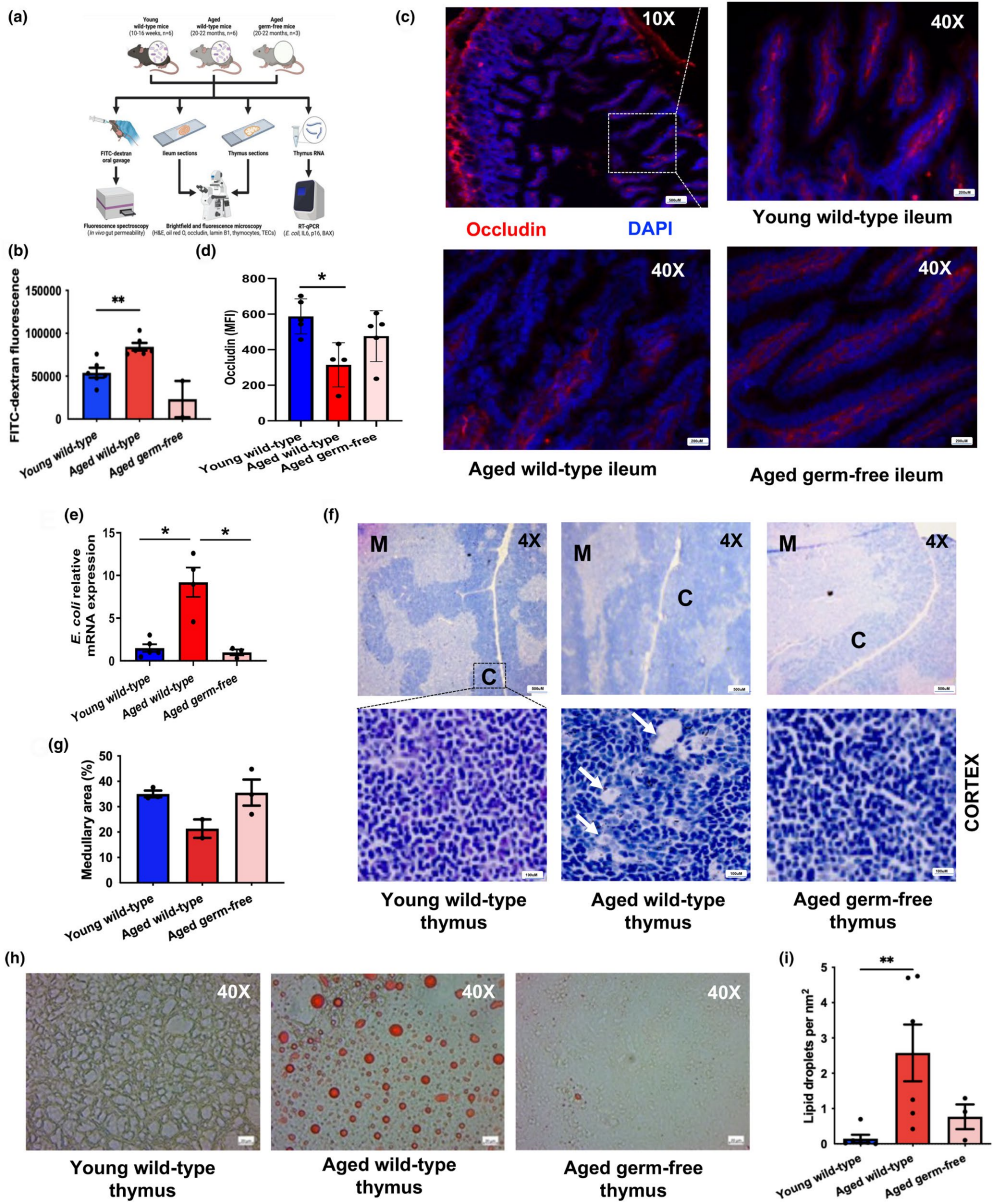
Thymic tissue organisation and adiposity in aged germ‐free mice protected from intestinal barrier dysfunction. (a) Experimental design for the mouse model used. (b) Intestinal permeability in young (*n* = 6) and aged (*n* = 6) wild‐type mice and aged germ‐free mice (*n* = 3) measured via FITC‐dextran fluorescence in blood following oral gavage. (c) Representative immunohistochemical images of tight junction protein, occludin‐1 (red staining) and nuclei (blue staining) in intestinal villi of mouse ileum sections taken at 10X magnification (top row) and 40X magnification (bottom row). (d) Occludin expression levels (MFI) in the intestinal villi (e) Relative mRNA expression levels of E. coli in young (*n* = 5) and aged (*n* = 4) wild‐type and aged germ‐free (*n* = 3) mouse thymuses. (f) Representative H&E stained images of mouse thymus sections with medullary regions stained light purple (indicated by M) and cortical regions stained dark purple (indicated by C). (g) Percentages of medullary regions in thymuses from young wild‐type (*n* = 3), aged wild‐type (*n* = 3) and aged germ‐free (*n* = 3) mice. Images on the top row were taken at a 4X magnification, and a higher 40X magnification was used for the cortex region shown on the bottom row. Arrows point to vacuoles. (h) Representative immunohistochemical images of oil red O stained mouse thymus sections at 40X magnification. (i) The number of lipid droplets per μm^2^ in young and aged wild‐type mice (*n* = 6 for both) and aged germ‐free mice (*n* = 3). Statistical significance was determined using one‐way ANOVA followed by Dunns’ multiple comparison test. *p ≤ 0.05, **p ≤ 0.01.

To determine whether an age‐related increase in circulating microbial products drives thymic involution, we used an aged germ‐free mouse model that was previously shown to be protected from increased gut permeability (Thevaranjan et al., 2017). We hypothesised that these aged germ‐free mice (20–22 months) would also be protected from key features of thymic involution (Figure 4a). The FITC‐dextran assay is a well‐established method for measuring in vivo intestinal permeability (Voetmann et al., 2023). In this study, we confirmed that aged wild‐type mice display enhanced translocation of FITC‐dextran into the blood compared to young wild‐type mice (*p* = 0.004), whereas aged germ‐free mice maintained intestinal barrier function (*p* = 0.14) (Figure 4b). Whilst there was no gross difference in intestinal architecture, we found that aged wild‐type mice exhibit decreased expression of the tight junction protein occludin compared to young wild‐type and aged germ‐free mice (Figure 4c). Additionally, aged germ‐free mice that were protected from intestinal membrane permeability displayed lower relative mRNA expression levels of *Escherichia coli* in the thymus compared to aged wild‐type mice (*p* = 0.03), which exhibited increased thymic *E. coli* mRNA expression levels relative to young mice (*p* = 0.01) (Figure 4d).

Age‐related thymic involution disrupts the structural integrity and cellular architecture of the thymus, resulting in the shrinkage of medullary regions and impaired thymopoiesis (Aw et al., 2008). Additionally, the corticomedullary junction, which separates the cortex and medulla and serves as a site for progenitor (CD4^−ve^CD8^−ve^) immigration and naïve T cell emigration (Nitta & Takayanagi, 2021), becomes disorganised with age and there is increased thymic adiposity (Palmer, 2013). In this study, the morphological analysis showed medullary shrinkage (Figure 4e), disruption of the corticomedullary junction and the appearance of vacuoles in aged wild‐type mouse thymuses, but not in those from aged germ‐free mice (Figure 4f). Oil‐red O staining, which is used for the enumeration of lipid‐laden tissues, also revealed an increase in the size and number of lipid deposits in the cortex and medullary regions of thymuses of aged wild‐type mice (*p* = 0.01) compared to young mice, whereas aged germ‐free mice were protected from lipid accumulation (*p* = 0.06) (Figure 4g,h).

**FIGURE 5:**
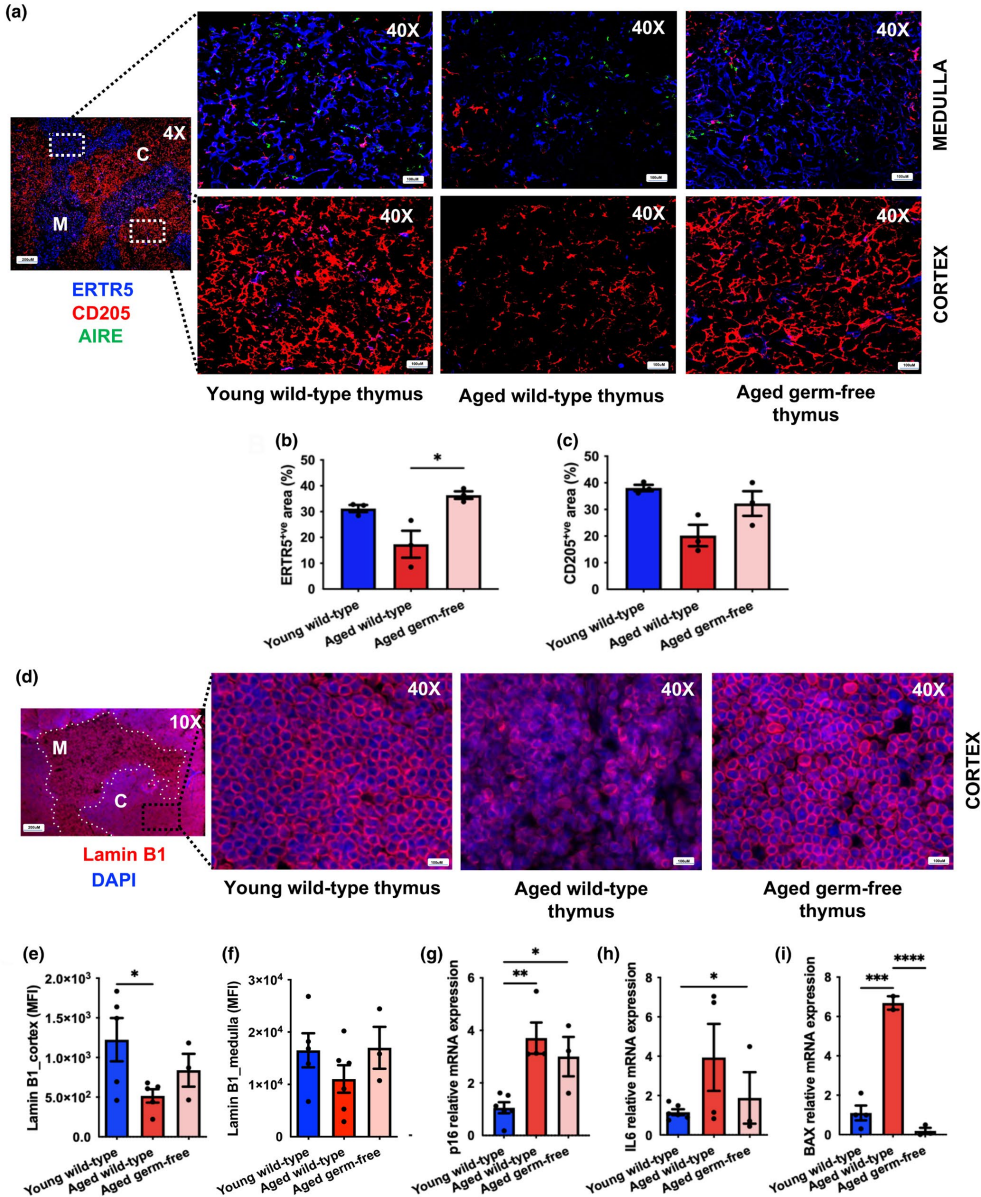
Thymic involution and underlying mechanisms in aged wild‐type and aged‐germ free mice. (a) Representative mouse thymus images depicting the distribution of CD205^+ve^ cTECs (red), ERTR5^+ve^ mTECs (blue) and AIRE^+ve^ mature mTECs (green) at 4X magnification (left) and 40X magnification (right). Percentages of ERT5^+ve^ areas (b) and CD205^+ve^ areas (c) in thymuses from young wild‐type (*n* = 3), aged wild‐type (*n* = 3) and aged germ‐free (*n* = 3) mice. (d) Lamin B1 (red) and DAPI (blue) stained mouse thymus sections imaged at 4X magnification (left) and 40X magnification (right). Medullary islands are marked by ‘M’ and dotted lines, while cortical regions are marked by ‘C’. Lamin B1 thymic expression levels (MFI) in the cortex (e) and medulla (f). Relative mRNA expression levels of p16 (g) , IL6 (h) and BAX (i) in thymuses from young wild‐type (*n* = 6), aged wild‐type (*n* = 6) and aged germ‐free (*n* = 3) mice. Statistical significance was determined using one‐way ANOVA followed by Dunns’ multiple comparison test. *p ≤ 0.05, **p ≤ 0.01, ***p ≤ 0.001, ****p ≤ 0.0001.

Stromal components of the thymus include specialized TECs that provide signals that induce the development and functional maturation of T lymphocytes. However, the aged thymus displays an enlargement of non‐cellular perivascular spaces and TEC reductions (Gui et al., 2007). Although perivascular spaces enable easy trafficking of cells and soluble proteins through the medulla, an increase in non‐cellular space permits the infiltration of pro‐inflammatory adipocytes and circulating senescent cells in the aged thymus. Morphological analysis revealed that CD205^+ve^ cortical and ERTR5^+ve^ medullary epithelial regions shrink and become less dense with age followed by the appearance of epithelial‐free areas in aged wild‐type mouse thymuses, though this did not occur in aged germ‐free mice (Figure 5a–c).

Next, we assessed the presence of senescent cells in the thymus of aged wild‐type and germ‐free mice to elucidate a potential mechanism underpinning the changes in thymus architecture. Lamin B1 is a structural protein that supports TEC development and maintains thymic architecture (Yue et al., 2019), and reduced lamin B1 expression is a marker of cellular senescence and thymic involution (Freund et al., 2012). Aged wild‐type mice exhibited tissue disorganisation and fewer lamin B1‐expressing cells in the cortex compared to young mice, but these ageing features were not present in aged germ‐free mice (Figure 5d). Further, we observed lower lamin B1 expression levels (mean fluorescent staining intensity (MFI)) in the cortex of aged wild‐type mice (*p* = 0.04), but not in aged germ‐free mice (*p* = 0.57), compared to young wild‐type mice (Figure 5e). In contrast, lamin B1 expression levels (MFI) in the medulla were unaffected by ageing (*p* = 0.22) or microbiota composition (*p* = 0.1) (Figure 5f). Transcriptional profiling of aged wild‐type mouse thymuses revealed increased mRNA expression levels of the cellular senescence marker p16 relative to young mice (*p* = 0.01), which were even lower in aged germ‐free mice (*p* = 0.04) (Figure 5g).

Elevated IL6 levels found in the thymuses of aged mice are associated with poorer thymic function and involution (Sempowski et al., 2000). However, aged germ‐free mice were previously shown to be protected from inflammageing, lacking high circulating levels of IL6 (Thevaranjan et al., 2017). Here we confirm that compared to young mice, aged wild‐type mice exhibit increased IL6 mRNA expression levels in the thymus (*p* = 0.05), which were lower in aged germ‐free thymuses (*p* = 0.55) (Figure 5h). Compared to young mice, mRNA expression levels of the apoptosis‐promoting gene BCL2 associated X (BAX) were also significantly higher in aged wild‐type mice (*p* = 0.0001) but not in aged germ‐free mice (*p* < 0.0001) (Figure 5i), suggesting a possible mechanism by which increased apoptosis drives TEC loss with age.

**FIGURE 6:**
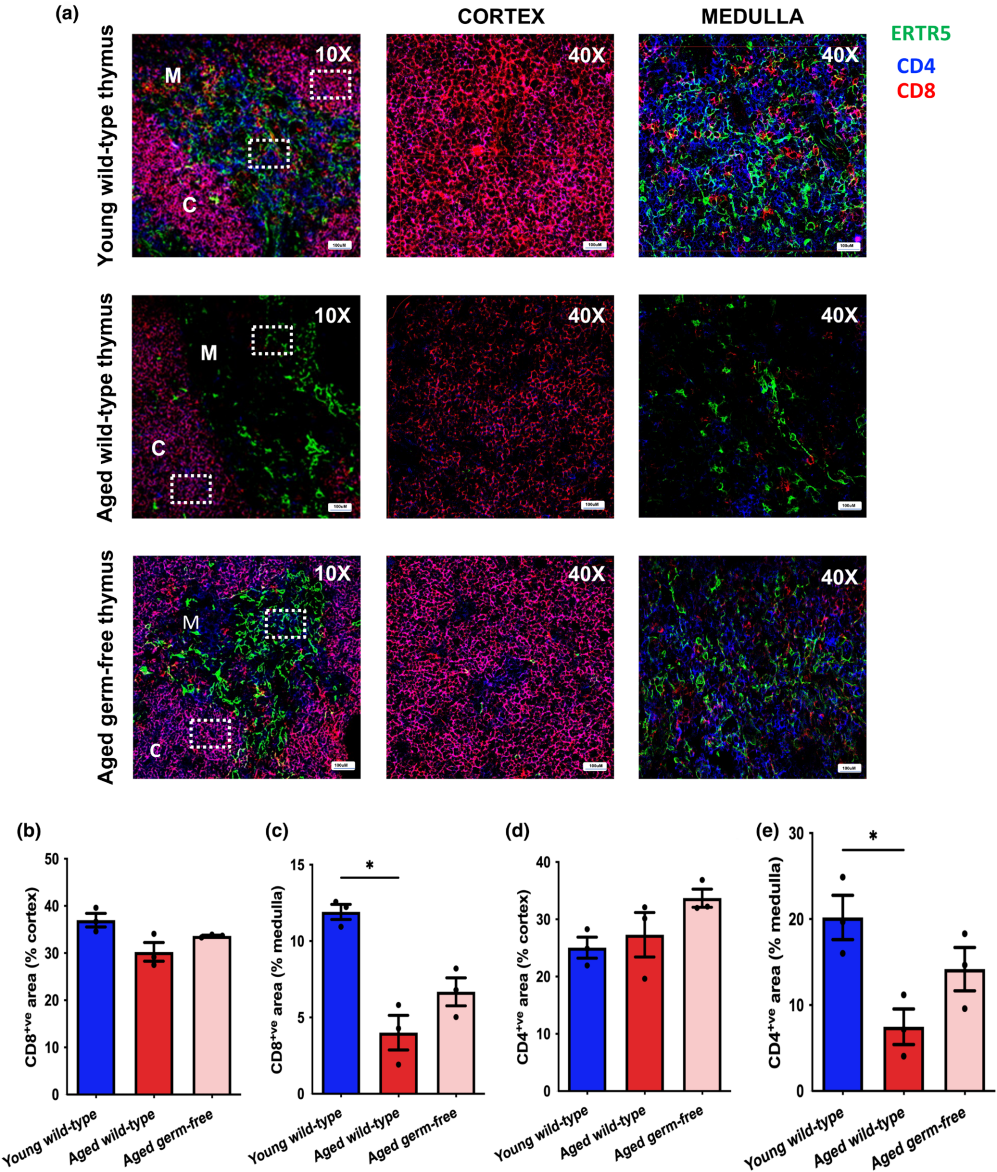
Thymocyte distribution in aged wild‐type and aged germ‐free mice. (a) Mouse thymus sections stained with CD4 (blue), CD8 (red) and ERTR5 (green) to assess the distribution of thymocytes in ERTR5^−ve^ cortical (marked as ‘C') and ERTR5^+ve^ medullary (marked as ‘M') regions. Images on the left were taken at 10× magnification, while images on the right were taken at 40× magnification. Percentages of CD8^+ve^ areas in the (b) cortex and (c) medullary regions in thymuses from young wild‐type (*n* = 3), aged wild‐type (*n* = 3) and aged germ‐free (*n* = 3) mice. Percentages of CD4^+ve^ areas in the (d) cortex and (e) medullary regions in thymuses from young wild‐type (*n* = 3), aged wild‐type (*n* = 3) and aged germ‐free (*n* = 3) mice. Statistical significance was determined using one‐way ANOVA *p ≤ 0.05.

Lastly, we examined the distribution of CD4 and CD8 expressing thymocytes in the thymus, where CD4^+^CD8^+^ cells would typically reside in cortical areas while more mature CD4^+^CD8^−^ and CD4^−^CD8^+^ would reside in medullary areas. While CD4^+^CD8^+^ and single positive CD4^+^ and CD8^+^ thymocytes were distributed normally within thymus tissue, we found that aged wild‐type mice display a loss of CD4^+^CD8^+^ cortical regions in the thymus (Figure 6a). However, the proportion of CD8‐expressing thymocytes is higher in aged germ‐free mice compared to aged wild‐types in medullary regions (Figure 6c). A similar decline in the proportion of CD4‐expressing thymocytes was also observed in the medulla with age, though aged germ‐free mice displayed a higher proportion of CD4^+ve^ areas (Figure 6e).

## 3 DISCUSSION

It is becoming increasingly clear that impaired intestinal barrier integrity is a major pathophysiological feature of ageing that contributes to the decline of organismal health. To the best of our knowledge, this study is the first to report an age‐associated increase in intestinal membrane permeability and systemic microbial translocation in healthy aged individuals in line with findings from non‐human primate studies (Tran & Greenwood‐Van Meerveld, 2013). Dietary components play an integral role in modulating intestinal barrier integrity. For instance, there is mounting evidence that excessive consumption of dietary fats enhances intestinal membrane permeability (Gulhane et al., 2016), predisposing individuals to local and systemic inflammation. Interestingly, high adherence to the MedDiet and consumption of a high‐quality diet were inversely correlated with intestinal barrier dysfunction in this study, possibly due to the anti‐inflammatory and health‐promoting properties of dietary fibre. This is supported by a study that reported improvements in intestinal barrier integrity in middle‐aged women following high adherence to the MedDiet (Seethaler et al., 2023).

Changes in gut microbiota composition with age are also closely linked with the onset of intestinal barrier leakage in mice. Accordingly, intestinal barrier leakage was correlated with higher relative abundances of pro‐inflammatory *Escherichia‐Shigella*, *Peptostreptococcaceae* and *Paraprevotella* in stool. On the other hand, robust intestinal barrier integrity was positively associated with high faecal levels of propionate and GDCA, both of which exert immunomodulatory and anti‐inflammatory effects on the immune system.

In this study, we propose that age‐related microbial translocation induces a state of persistent T‐cell activation. These results are supported by an earlier study that reported loss of gut mucosa homeostasis and increased bacterial translocation in HIV patients, resulting in chronic immune system activation and systemic inflammation (Brenchley et al., 2006). Therefore, we hypothesise that persistent stimulation caused by microbial translocation promotes the terminal differentiation of T cells and induces cellular senescence in aged T cells, together accelerating immune ageing. These observations are in line with those from another study reporting close links between microbial translocation and memory T cell expansion in adult mice (Cruz‐Adalia et al., 2017).

In agreement with other ageing studies (Pieren et al., 2019), we observed an age‐related increase in the percentage of Tregs that was correlated with increased gut permeability. There is emerging evidence of a potential link between the expansion of senescent T cells and Tregs, with studies demonstrating that Tregs trigger DNA damage in effector T cells via metabolic competition during cross‐talk, resulting in cellular senescence and functional exhaustion (Liu et al., 2018). Thus, we propose that increased microbe recognition caused by enhanced bacterial translocation might contribute to dysregulated ROS production and altered glucose metabolism in aged Tregs, promoting aberrant Treg interactions and senescent cell accumulation.

Consistent with the findings discussed above, we identified several hallmarks of ageing in circulating immune cells, including upregulation of pro‐inflammatory signalling markers (HMBG1), defective autophagy processes (ATG7 and LAMP), reduced DNA damage repair (ATM), increased cellular senescence (gain of KLRG1 and loss of CD28), enhanced apoptosis (BCL2, CASP3 and CASP8), loss of proliferation regulators (DUSP4 and DUSP6) and upregulation of cell‐cycle arrest regulators (TXNIP), that were only present in older adults with high microbial translocation. These results are in line with those from a study that demonstrated that microbial products disrupt autophagosome formation and trigger mitochondrial dysfunction by interfering with Rab1A signalling and reducing mitochondrial coupling (Feng et al., 2018; Tzika et al., 2013).

In this study, we report a significant age‐related loss of RTEs in older adults with high MT, supporting our hypothesis that circulating bacterial products have deleterious effects on thymopoiesis. To confirm that microbial products contribute towards age‐related thymic involution, we used aged germ‐free mice, which are protected from loss of intestinal barrier function. Here, we demonstrate for the first time that ageing is accompanied by increased thymic translocation of *E*. *coli* in wild‐type mice but not in germ‐free mice. Importantly, hallmarks of thymic involution, including the loss of functional thymic niches due to the depletion of TECs, adipocyte infiltration and senescent cell accumulation, were less pronounced in aged germ‐free mice.

In vitro analysis reveals that LPS, found on the surface of gram‐negative bacteria such as *E*. *coli*, promotes the accumulation of lipid droplets in endothelial cells (Czamara et al., 2021), induces cellular senescence and enhances the SASP of senescent cells (Kim et al., 2012). Thus, elevated circulating levels of microbes and microbial products, like LPS, could promote increased thymic adiposity and cellular senescence in aged hosts. Accumulation of senescent cells and adipocytes during ageing is believed to hinder thymic function through increased secretion of pro‐inflammatory cytokines, such as IL6 and TNFα. In this study, ageing was accompanied by increased thymic expression of IL6 in wild‐type mice. However, aged germ‐free mice exhibited comparable IL6 expression levels to those in young wild‐type mice, indicating a role for microbial translocation in the age‐dependent upregulation of thymopoiesis‐suppressing cytokines. Indeed, LPS treatment and *E*. *coli* enterotoxin cause thymic atrophy, leading to the loss of single positive (CD4^−ve^CD8^+ve^ and CD4^+ve^CD8^−ve^) thymocytes as well as double positive (CD4^+ve^CD8^+ve^) and double negative (CD4^−ve^CD8^−ve^) thymocytes (Majumdar et al., 2019). One mechanism by which this occurs is through LPS‐induced apoptosis of thymocytes (Zhang et al., 1993). Supporting this, thymic expression of the apoptotic gene BAX increased with age in wild‐type mice, whereas aged germ‐free mice were unaffected.

Although therapeutic manipulation of the gut microbiota might improve health in aged hosts, it remains unclear how restoring intestinal barrier function possibly by targeting microbiome dysbiosis could reverse features of immune ageing. For instance, studies have reported links between microbial composition, intestinal membrane permeability and circulating cytokine levels in aged hosts, but have not investigated their impact on immune health (Claesson et al., 2012). Our data demonstrates that transferring healthy gut microbiota into *Clostridium difficile* infected older adults is sufficient to improve intestinal barrier integrity. Moreover, faecal microbiota transplantation promotes the expansion of peripheral naïve T cells and reduces the senescent T cell burden in recipients, suggesting potential anti‐immunesenescence effects (Monaghan et al., 2021). Microbial dysbiosis in HIV patients, characterised by the loss of beneficial *Bifidobacterium* and the overrepresentation of *Clostridium* clusters, is also alleviated in response to probiotic administration, resulting in reduced microbial translocation and improved immune cell function (Gori et al., 2011). The appearance of opportunistic microbial communities in the aged gut is related to dietary changes, such as low consumption of fibre‐rich fruits and vegetables and increased consumption of meats and processed foods (Claesson et al., 2012). Moreover, studies have reported rebalancing of the gut flora, reduced systemic inflammation and improved health status in older adults who consume a MedDiet (Ghosh et al., 2020). Therefore, high adherence to a MedDiet rich in fibre, polyunsaturated fats, minerals and vitamins could strengthen gut barrier function and improve immune function in the elderly.

This study has a few limitations, the first being that the exact mechanisms linking intestinal membrane permeability to immune ageing remain to be fully elucidated. Secondly, although we report an increase in microbial translocation with age, we do not know the nature of these microbial products. Thus, further work is required to determine the impact of individual bacterial products on immune ageing.

Like all research studies, ours has a few limitations that should be noted. Firstly, our use of strict inclusion criteria excluded older adults with any underlying comorbidities, immune‐mediated diseases and gastrointestinal disorders. Our cohort of older adults, who were interested in biogerontology research and keen to partake in our study, are all extremely healthy, consume a high‐quality diet rich in dietary fibres and engage in regular physical activity (only one individual was sedentary). Unfortunately, this might not be a true representation of the ageing population, which is considered to be malnourished, largely sedentary and ridden with multimorbidity. However, this strategy dissected the intrinsic effects of ageing and highlighted the novel interactions that we observed are features of intestinal barrier dysfunction and T‐cell ageing. However, in a current ongoing study, we are addressing this by recruiting older individuals with underlying comorbidities to identify immune‐intestinal barrier signatures and interactions that differ between individuals on healthy vs. unhealthy ageing trajectories. Another key limitation is that our results are based on a cohort of Caucasian participants, and we would like to validate our findings in a larger study (enabling us to dissect sex differences) conducted on older adults with more ethically and geographically diverse backgrounds.

In conclusion, age‐related thymic involution is a known hallmark of T cell ageing that contributes significantly toward immunesenescence. Nevertheless, we suggest that systemic microbial translocation due to increased intestinal barrier leakage contributes towards a reduced thymic output and the emergence of T cell ageing features Thus, our findings advocate for targeting intestinal barrier integrity as a novel strategy for promoting thymic rejuvenation and combating T cell ageing in the elderly. Exploiting the restoration ability of these targets provides new opportunities to cope with lagging health span developments of individualised dietary, probiotic and postbiotic interventions.

## 4 METHODS

### 4.1 Participants and study design

This is an observational, cross‐sectional study in healthy young (aged 18–37 years) and 55 healthy old (aged ≥60 years) adults, the latter of whom were enrolled from the Birmingham 1000 Elders group, were recruited into this study from November 2019 to December 2020. The exclusion criteria for healthy participants included self‐reported infections, comorbidities (e.g. chronic inflammatory/autoimmune conditions and cancer), use of immunosuppressants, antibiotic usage in the past 2 months, hospitalisation in the past 3 months and/or having travelled outside of the UK in the month leading up to recruitment. Written informed consent was obtained from all eligible volunteers prior to sampling and additional participant demographics, including height, weight, BMI, physical activity levels (hours of TV watched and stairs climbed), sleep quality, mental health status (anxiety and depression) and diet, were gathered via self‐assessed questionnaires (Figure 1a). Blood samples (30 mL) were collected from all participants between 9:00–11:00 am in BD vacutainers containing lithium heparin. Complete blood cell counts were obtained using a Sysmex XN‐1000 automated haematology analyser (Sysmex).

### 4.2 Isolation and freezing of peripheral blood mononuclear cells (PBMCs)

PBMCs were isolated from whole blood samples via density gradient centrifugation using Ficoll‐Paque™ PLUS density gradient media (GE Healthcare). Isolated PBMCs were frozen by resuspending the cells in freezing medium consisting of 10% dimethyl sulfoxide (Sigma Aldrich) in heat‐inactivated fetal calf serum (HiFCS) (Biosera) and stored at −80°C.

### 4.3 Serum microbial translocation and cytokine analysis

Blood collected in anti‐coagulant free BD vacutainers was left upright for 30 min prior to centrifugation at 1620 × *g* for 10 min at room temperature, after which the serum was removed and stored at −80°C prior to analysis. The concentration of serum proteins (occludin, LBP, sCD14 and sCD25) and cytokines (IL1β, IL4, IL6, IL7, IL10, IL15, IL17, TNFα, IFNγ, CRP, CXCL9 and GM‐CSF) was determined using commercially available Enzyme‐Linked Immunosorbent Assays (DL Develop, R&D Systems and Invitrogen) and a Human Premixed Multi‐Analyte Magnetic Luminex Assay (R&D Systems). The absorbance of the wells was measured using wavelengths specified by the manufactures' instructions on a spectrophotometer (BioTek). Protein and cytokine concentrations were extrapolated from a standard curve created using known concentrations via GraphPad Prism software v9 (GraphPad Software Inc.).

### 4.4 Stool 16S rRNA gene amplification via RT‐qPCR

DNA was extracted from 500 mg aliquots of stool using a commercially available FastDNA™ SPIN Kit for Soil (MP Biomedicals) according to the manufacturer's instructions. DNA concentrations were determined on a calibrated Qubit 4 Fluorometer (Invitrogen™). RT‐qPCR was used to amplify the V4 region of the 16S rRNA gene using desalted 515F and 806R primer pairs (Sigma‐Aldrich) to create an amplicon library. Stool DNA samples (0.16–14.48 ng/μL) were amplified in triplicate under the following PCR thermocycler conditions using a SensoQuest Basic Thermal Labcycler (with gradient; Geneflow Ltd., UK): initial denaturation at 94°C for 3 min followed by 35 cycles of denaturation at 94°C for 45 s, annealing at 50°C for 60 s, elongation at 72°C for 90 s and an extended elongation step at 72°C for 10 min. After pooling triplicate PCR reactions, RT‐qPCR products were purified using the GeneJET PCR Purification Kit (Thermo Scientific™) according to the manufacturer's instructions. The quality of the DNA library was checked via TapeStation D1000 ScreenTape® (Agilent Technologies) using TapeStation Analysis Software A.02.02 (SR1) (Agilent Technologies). High‐thorough 16S rRNA sequencing on the Illumina MiSeq platform (Illumina, Inc.) was done by Genomics Birmingham (University of Birmingham) using pooled DNA library and the MiSeq v2 500 sequencing kit (Illumina, Inc.). 16S rRNA sequencing data was analysed on the web‐based Galaxy platform (version 23.01.dev0) using the Mothur toolset according to the online‐based tutorial, unique sequences were aligned to the SILVA v132 reference alignment to improve clustering of the operational taxonomic units (OTUs), using the Needleman‐Wunsch algorithm. Next, taxonomic classification was performed using the SILVA v132 reference taxonomy and the Bayesian classifier and bacterial sequences were clustered into OTUs at the phylum level using a 97% similarity threshold and the relative abundance (%) of each phylum and genus was calculated.

### 4.5 Liquid chromatography‐mass spectrometry

Faecal SCFA (acetate, butyrate and propionate) and secondary bile acid (deoxycholic acid (DCA), glycodeoxycholic acid (GDCA), hyodeoxycholic acid (HDCA), lithocholic acid (LCA) and ursodeoxycholic acid (UDCA)) concentrations were assessed in all participants via liquid chromatography‐mass spectrometry (LC–MS) performed by the Quadrum Institute (Norwich, UK). In preparation, stool samples were thawed and 1 mL of extraction solvent (0.5% (*v*/*v*) orthophosphoric acid) was added to each tube followed by centrifugation and passing the supernatants through a Mini‐UniPrep filter vial (0.45 μm; Whatman plc) prior to SCFA analysis via LC–MS. For the analysis of secondary bile acids, thawed stool (100 mg) was homogenised in a MaxQ™ 6000 incubated shaker (Thermo Fischer Scientific) at 500 rpm for 30 min at 20°C using 5–10 ceramic beads and 1 mL of methanol: water (9:1, *v*/*v*). Afterwards, 25 μL of 40 μg/mL D4‐glycocholic acid was added to 500 μL of isolated stool supernatant. The mixtures were passed through a 10 mg capacity Oasis PriME HLB 96‐well cartridge (Waters™) to remove phospholipids before LC–MS.

LC–MS was carried out on purified faecal samples and stool supernatant samples alongside a set of internal standards (D4‐acetic acid, D2‐propionic acid and D4‐ glycocholic acid) using an ACQUITY ultra‐high‐performance liquid chromatography system (Waters™) coupled to a Xevo TQ‐S micro triple quadrupole mass spectrometer (Waters™). The mass spectrometer was operated in positive multiple reaction mode for SCFA quantification, while negative electrospray selected ion monitoring mode was used for D4‐glycocholic acid (*m*/*z* 468.2), DCA and UDCA (*m*/*z* 391.2) and LCA (*m*/*z* 375.2) during secondary bile acid analysis.

### 4.6 T cell phenotyping

Frozen PBMCs were surface stained with a combination of anti‐human monoclonal antibodies for a 20 min incubation at 4°C in the dark: 2 μg/mL CD3‐PEcy7 (clone UCHT1; eBioscience™), 5 μg/mL CD4‐eFluor 450 (clone OKT4 (OKT‐4); eBioscience™), 3 μg/mL CD45RA‐PerCP/cy5.5 (clone HI100; Biolegend) and 8 μg/mL CCR7‐APCcy7 (clone G043H7; Biolegend), 4.5 μg/mL PTK7‐PE (clone 188B; Miltenyi Biotec), 8 μg/mL CD25‐APCcy7 (clone M‐A251; BD Pharmingen), 0.5 μg/mL CD28‐APC (clone CD28.2; eBioscience™), 6 μg/mL CD57‐FITC (clone TB01 (TB01); Biolegend), 4 μg/mL PD1‐APCcy7 (clone EH12.2H7; Biolegend) 0.5 μg/mL CD69‐PEcy5 (clone FN50; Biolegend) and 16 μg/mL CD154‐APCcy7 (clone 24–31; Biolegend). Post incubation, the cells were fixed, permeabilised and intracellularly stained with 4 μg/mL anti‐human RORγ‐APC antibody and 10 ng/mL anti‐human Foxp3‐PE antibody (clone PCH101; eBioscience™) using the Foxp3 Transcription Factor Staining Buffer Set (eBioscience™) to examine the frequency of peripheral Th17 cells and Tregs. Flow cytometric analysis was carried out on a MACSQuant Analyzer 10 benchtop flow cytometer (Miltenyi Biotec). Prior to data acquisition, concentration‐matched isotype controls were used to set the gates, and fluorescence spectral overlap was corrected via compensation during multi‐colour flow cytometry. Data analysis was performed using FlowJo software v10.8.1 (BD). T cells were defined as CD3^+ve^ cells and 10,000 cells were gated and divided into CD4^+ve^ and CD8^+ve^, which were further divided into four subsets based on CD45RA and CCR7 expression and denoted as naive (CD45RA^+ve^CCR7^+ve^), central memory (CD45RA^−ve^CCR7^+ve^), effector memory (CD45RA^−ve^CCR7^−ve^) and EMRA (CD45RA^+ve^CCR7^−ve^) (see Figure S1 for gating strategy). CD3^+ve^CD28^−ve^CD57^+ve^ cells were denoted as senescent T cells.

### 4.7 T cell functional analysis

Thawed PBMCs were washed with RPMI‐1640 medium supplemented with 10% heat inactivated foetal calf serum (HiFCS), 2 mM L‐glutamine, 100 U/mL penicillin and 100 μg/mL streptomycin (300 × *g* for 10 min at 20°C). Post centrifugation, the cells were incubated with Benzonase® Nuclease (Sigma‐Aldrich) for 1 h at 37°C with 5% CO_2_ before being transferred to the wells of a 96‐well U‐bottom plate pre‐coated with 0.5 μg/mL anti‐human CD3 monoclonal antibody (clone UCHT1; eBioscience™) and 5 μg/mL anti‐human CD28 monoclonal antibody (clone CD28.2; eBioscience™). Following a 20 h incubation at 37°C, 5% CO_2_, 10 μg/mL brefeldin A from *Penicllium brefeldianum* (Sigma‐Aldrich), 50 ng/mL phorbol 12‐myristate 13‐acetate (Sigma‐Aldrich) and 500 ng/mL ionomycin from *Streptomyces conglobatus* (Sigma‐Aldrich) were added to the wells and the PBMCs were incubated for an additional 4 h. Post incubation, the cells were washed with phosphate buffered saline (PBS) and stained with LIVE/DEAD™ Fixable Near‐IR Dead Cell Stain (Invitrogen) and 2 μg/mL CD3‐PEcy7 (clone UCHT1) for 20 min in the dark at 4°C. The PBMCs were then washed with PBS prior to being fixed and permeabilised using the Foxp3 Transcription Factor Staining Buffer Set. To examine IL10‐producing Tregs, the cells were stained with 2 μg/mL anti‐human CD4‐BV421 (clone RPA‐T4; BD Biosciences), 10 ng/mL anti‐human Foxp3‐PE (clone PCH101) and 100 ng/mL anti‐human IL10‐APC (clone JES3‐19F1; Biolegend) for 30 min in the dark at room temperature. Then, the PBMCs were washed and resuspended in 300 μL of PBS. Functional T cell data was assessed using FlowJo software. The percentage positive cells and MFI values were recorded for each antigen.

### 4.8 RNA isolation from PBMCs

RNA was isolated from 2 million frozen PBMCs according to the instructions of a commercially available RNeasy Mini Kit (Qiagen) and the purified RNA was eluted into 15 μL of RNase‐free water. RNA quantity and quality were determined using a NanoDrop One Spectrophotometer (Thermo Fischer Scientific) and a 2100 Bioanalyser (Agilent Technologies), respectively. RNA samples were considered pure if the A260/280 and A260/230 ratios were ≥1.8. Purified RNA samples were stored at −80°C for further analysis.

### 4.9 NanoString nCounter® gene expression analysis

NanoString nCounter® was utilised for multiple gene expression profiling in RNA samples using the nCounter® Human PanCancer Immune Profiling Panel, consisting of 729 genes related to cytokine and chemokine signalling, cellular senescence, immune cell profiling, lymphoid cell function and innate and adaptive immune responses (Nanostring Technologies). 80 ng of unamplified RNA per sample was processed by the Birmingham Tissue Analytics at the University of Birmingham. Normalization and data analysis of count numbers were carried out with NanoString nSolver® Analysis. Qlucore Omics Explorer software v3.8 (Qlucore) was used to create a heatmap using log_2_ fold‐change values to visualise unique gene expression patterns between cohorts. Genes were included if *p*‐values were statistically significant.

### 4.10 Mouse experiments

Male and female young (10–16 weeks) and aged (20–22 months) wild‐type C57BL/6 mice (originally from Jackson Laboratories) were fed a low protein diet (i.e. Teklad Irradiated Global 14% protein Maintenance Diet) and bred in‐house. Sex and age‐matched germ‐free mice (20–22 months) were housed in pathogen‐free conditions in the Gnotobiotic Facility of McMaster. All experiments were performed in accordance with the Institutional Animal Utilization protocols [protocol number 21‐04‐13] approved by McMaster University's Animal Research Ethics Board as per the recommendations of the Canadian Council for Animal Care. Thymus and ileum tissues were collected from young wild‐type (*n* = 6), aged wild‐type (*n* = 6) and aged germ‐free mice (*n* = 3).

### 4.11 FITC‐dextran trans‐epithelial intestinal permeability assay

Mice were fasted (no food or water) for 6 h prior to oral gavage of 150 μL of 80 mg/mL tracer labelled FITC‐dextran (Sigma‐Aldrich) to assess in vivo intestinal permeability. After 4 h, blood was collected and diluted 2‐fold with PBS. Fluorescent intensity was measured on a SpectraMax i3 microplate reader (Molecular Devices, USA) with an excitation wavelength of 493 nm and an emission wavelength of 518 nm.

### 4.12 Occludin staining for intestinal membrane permeability assessment

Sections of ileum were excised and embedded in OCT compound at −80°C. Tissue blocks were cut into 7 μm sections that were fixed with ice‐cold acetone. After blocking with 10% goat serum, the samples were stained with 4 μg/mL mouse anti‐mouse occludin antibody (clone E‐5; Santa Cruz Biotechnology) overnight, followed by incubation with 40 μg/mL goat anti‐mouse Alexa Fluor® 555 secondary antibody (Thermo Fischer™). Images were acquired using an Olympus IX71 inverted fluorescence microscope at 10× and 40× magnification.

### 4.13 Histological analysis and oil red O staining of mouse thymus sections

Frozen mouse thymuses embedded in OCT compound were sectioned to a thickness of 7 μm and mounted onto microscope slides before staining with haematoxylin and eosin (H&E) and the percentage of medullary areas was calculated as a percentage. Thymus sections were also stained for lipid droplets using an Oil Red O Staining Kit. Each thymus sample was stained in triplicate and six images were acquired using a Zeiss Primovert inverted light microscope at 10× and 40× magnification. The number of oil red O stained lipid droplets per μm^2^ was determined using Fiji software.

### 4.14 Nuclear lamin B1 staining

Microscope slides containing mouse thymus sections were fixed with ice‐cold acetone and stained with recombinant anti‐mouse lamin B1 antibody (clone EPR22165‐121; Abcam) consisting of 10% HiFCS and 0.2% triton‐X 100 in PBS overnight followed by staining with 6.6 μg/mL anti‐rabbit IgG conjugated to Alexa Fluor® 555 (clone H + L, F(ab’)_2_; Cell Signalling Technology). Post wash, tissues were stained with 1 μg/mL DAPI solution (Thermo Fischer™). Images were acquired using an Olympus IX71 inverted fluorescence microscope at 10 and 40× magnification and imaging analysis was performed using Image J software.

### 4.15 Thymocyte and thymic epithelial cell (TEC) staining

Frozen thymus sections fixed with ice‐cold acetone were stained with a combination of anti‐mouse monoclonal antibodies for 30 min in the dark at room temperature: 2.5 μg/mL CD4 Alexa Fluor 647 (clone RM4‐5; Biolegend), 1.7 μg/mL CD8α biotin (clone 53–6.7; eBioscience™), ERTR5 rat IgM, 10 μg/mL CD205 biotin (clone 205yeka; eBiosience™) and 10 μg/mL Aire Alexa Fluor 488 (clone 5H12; Thermo Fischer Scientific). Post incubation, the slides were incubated with the following secondary antibodies: 2 μg/mL streptavidin Alexa Fluor 555 (Thermo Fischer Scientific), 2.5 μg/mL goat anti‐rat IgM Alexa Fluor 488 (Thermo Fischer Scientific) and 10 μg/mL goat anti‐rat IgM Alexa‐Fluor 647 (Thermo Fischer Scientific). The slides were then washed and incubated with 1 μg/mL DAPI solution (Thermo Fischer™) in preparation for confocal microscopy. Each thymus sample was stained in triplicate and six images of the medullary and cortical regions were acquired on Zeiss LSM 880 with Airyscan Fast confocal microscope at 10 and 40× magnification. Before image acquisition, primary antibody‐only controls and secondary antibody‐only controls were used to detect possible non‐specific binding and autofluorescence. Confocal imaging analysis was performed using Zeiss Zen Black software to calculate the positively staining ERTr5 and CD205 pixels were expressed as a percentage of the total pixels in the picture.

### 4.16 RNA isolation and quantitative real time‐PCR

RNA was isolated from thymus samples using a commercially available RNeasy Mini Kit as per the manufacturer's instructions (Qiagen). RNA quantity and quality were determined using a NanoDrop One Spectrophotometer and a 2100 Bioanalyser.Quantitative. Quantitative real time‐PCR was carried out on 5 ng/μL RNA isolated from mouse thymus samples using the iTaq Universal SYBR Green One‐Step Kit (Biorad) on a CFX384 Tough Real‐Time PCR Detection System (Biorad). Primer sequences were p16 (forward primer TTGGCCCAAGAGCGGGGACA; reverse primer GCGGGCTGAGGCCGGATTTA), IL6 (forward primer CTGCAAGAGACTTCCATCCAG; reverse primer AGTGGTATAGACAGGTCTGTTGG), BAX (forward primer GTTTCATCCAGGATCGAGCAG; reverse primer CATCTTCTTCCAGATGGTGA), *E*. *coli* (forward primer GGTAGAGCACTGTTTTGGCA; reverse primer TGTCTCCCGTGATAACTTTCT) and housekeeping gene Epcam (forward primer TTGCTCCAAACTGGCGTCTAA; reverse primer GCAGTCGGGGTCGTACA). The PCR thermocycler condition was as follows: initial reverse transcription at 50°C for 10 min, polymerase activation at 95°C for 5 min, 40 cycles of denaturation at 95°C for 10 s, annealing at 60°C for 30 s and initial elongation at 65°C for 31 s followed by 60 cycles of elongation at 65°C for 5 s. All samples were run in triplicate. Relative gene expression was calculated using the ΔΔCt method followed by normalisation of the values to the relative gene expression of Epcam.

### 4.17 Statistical analysis

All statistical analysis was performed using GraphPad Prism® software (GraphPad Software Inc.). Data distribution was examined using Kolmogorov–Smirnov normality test before parametric and non‐parametric tests were performed. Parametric tests were carried out on normally distributed data, while non‐parametric tests were used when the data was not normally distributed. Unpaired Student's *t* test (parametric test) and Mann–Whitney *U* test (non‐parametric) were used to compare means between young and old adults and the Benjamini‐Hochberg method was used to calculate adjusted *p*‐values. Chi‐square test was used to compare categorical data, such as sex and smoking status, between the groups. One‐way analysis of variance (ANOVA) was used to compare means between low MT young, low MT old and high MT old adults as well as means between young wild‐type, aged wild‐type and aged germ‐free mice. Following one‐way ANOVA, Bonferroni (parametric test) and Dunn's (non‐parametric) multiple comparison tests were performed to calculate adjusted *p*‐values. Spearman correlation‐based linear regression analysis was performed to determine the strength of associations between all combinations of intestinal barrier dysfunction surrogate markers and hallmarks of immunesenescence. For pathway enrichment analysis, the Benjamin‐Hochberg false discovery rate was used to calculate adjusted *p*‐values. Statistical significance was accepted as *p* ≤ 0.05.

## AUTHOR CONTRIBUTIONS

N.A.D. conceptualised the study and secured funding. J.C. performed the recruitment, multi‐colour immunophenotyping on young and old participants and analysed the flow cytometry data. She also performed the sectioning of mice tissue and immunostainings on thymus sections. E.N.D.J. and D.W.E. designed and provided the resources for the animal experiments. B.D. performed the immune functional assays. N.R.P. performed the immunostainings on the gut tissue sections and PCRs. A.S.O. and J.C. supported the statistical analysis. A.J.W. has supported imaging of the stained tissue slides using the confocal microscope. N.P.R. isolated the RNA from thymus sections and performed the PCRs. L.E.L. supported J.C. with the 16S RNA sequencing. N.A.D. and J.C. wrote the first draft of the manuscript. G.A., D.M.E., C.M. reviewed and edited the manuscript. W.v.S. and L.E.L. have supported the 16S RNA sequencing. All authors read and approved the final manuscript.

## FUNDING INFORMATION

This work was supported by funding from the Academy of Medical Sciences Springboard Award (SBF0051132) and the MRC‐Versus Arthritis Centre for Musculoskeletal Ageing Research. GA is supported by an MRC Programme Grant to GA (MR/T029765/1). CM is supported by a BHF funded fellowship. The views expressed here are those of the authors and not necessarily those of the Department for Health and Social care. The funders provided financial support to this research but had no role in the design of the study, analysis, interpretations of the data and in writing the manuscript.

## CONFLICT OF INTEREST STATEMENT

The authors declare that they have no conflicts of interest connected to this paper.

## CONSENT FOR PUBLICATION

The manuscript does not contain any individual person's data in any form.

## Supporting information


Data S1:

## Data Availability

The raw flow cytometry data generated during the current study is available on Flow Repository http://flowrepository.org/id/RvFr2BwD439Kk8OTGwD82uOAa7rbYKEAdkFtjkN5lrRGaefOrmYPTryOvjZylV2O. The microscopy images generated during the current study is available https://docs.google.com/presentation/d/1pfYeYpOiT3jiClZaPMcZek9FMAojnAeiAHlfleZD5sU/edit?usp=sharing. All gene expression generated data is included in this published article [and its supplementary information files].

## References

[acel14401-bib-0001] Alpert, A. , Pickman, Y. , Leipold, M. , Rosenberg‐Hasson, Y. , Ji, X. , Gaujoux, R. , Rabani, H. , Starosvetsky, E. , Kveler, K. , Schaffert, S. , Furman, D. , Caspi, O. , Rosenschein, U. , Khatri, P. , Dekker, C. L. , Maecker, H. T. , Davis, M. M. , & Shen‐Orr, S. S. (2019). A clinically meaningful metric of immune age derived from high‐dimensional longitudinal monitoring. Nature Medicine, 25, 487–495. 10.1038/s41591-019-0381-y PMC6686855 30842675

[acel14401-bib-0002] Aw, D. , Silva, A. B. , Maddick, M. , von Zglinicki, T. , & Palmer, D. B. (2008). Architectural changes in the thymus of aging mice. Aging Cell, 7, 158–167. 10.1111/j.1474-9726.2007.00365.x 18241323

[acel14401-bib-0003] Brenchley, J. M. , Price, D. A. , Schacker, T. W. , Asher, T. E. , Silvestri, G. , Rao, S. , Kazzaz, Z. , Bornstein, E. , Lambotte, O. , Altmann, D. , Blazar, B. R. , Rodriguez, B. , Teixeira‐Johnson, L. , Landay, A. , Martin, J. N. , Hecht, F. M. , Picker, L. J. , Lederman, M. M. , Deeks, S. G. , & Douek, D. C. (2006). Microbial translocation is a cause of systemic immune activation in chronic HIV infection. Nature Medicine, 12, 1365–1371. 10.1038/nm1511 17115046

[acel14401-bib-0004] Claesson, M. J. , Jeffery, I. B. , Conde, S. , Power, S. E. , O'Connor, E. M. , Cusack, S. , Harris, H. M. , Coakley, M. , Lakshminarayanan, B. , O'Sullivan, O. , Fitzgerald, G. F. , Deane, J. , O'Connor, M. , Harnedy, N. , O'Connor, K. , O'Mahony, D. , van Sinderen, D. , Wallace, M. , Brennan, L. , … O'Toole, P. W. (2012). Gut microbiota composition correlates with diet and health in the elderly. Nature, 488, 178–184. 10.1038/nature11319 22797518

[acel14401-bib-0005] Cruz‐Adalia, A. , Ramirez‐Santiago, G. , Osuna‐Pérez, J. , Torres‐Torresano, M. , Zorita, V. , Martínez‐Riaño, A. , Boccasavia, V. , Borroto, A. , Martínez Del Hoyo, G. , González‐Granado, J. M. , Alarcón, B. , Sánchez‐Madrid, F. , & Veiga, E. (2017). Conventional CD4^+^ T cells present bacterial antigens to induce cytotoxic and memory CD8^+^ T cell responses. Nature Communications, 8, 1591. 10.1038/s41467-017-01661-7 PMC5691066 29147022

[acel14401-bib-0006] Czamara, K. , Stojak, M. , Pacia, M. Z. , Zieba, A. , Baranska, M. , Chlopicki, S. , & Kaczor, A. (2021). Lipid droplets formation represents an integral component of endothelial inflammation induced by LPS. Cells, 10, 1403. 10.3390/cells10061403 34204022 PMC8227392

[acel14401-bib-0007] Duggal, N. A. (2018). Reversing the immune ageing clock: Lifestyle modifications and pharmacological interventions. Biogerontology, 19, 481–496. 10.1007/s10522-018-9771-7 30269199 PMC6223743

[acel14401-bib-0008] Elyahu, Y. , Hekselman, I. , Eizenberg‐Magar, I. , Berner, O. , Strominger, I. , Schiller, M. , Mittal, K. , Nemirovsky, A. , Eremenko, E. , Vital, A. , Simonovsky, E. , Chalifa‐Caspi, V. , Friedman, N. , Yeger‐Lotem, E. , & Monsonego, A. (2019). Aging promotes reorganization of the CD4 T cell landscape toward extreme regulatory and effector phenotypes. Science Advances, 5, eaaw8330. 10.1126/sciadv.aaw8330 31457092 PMC6703865

[acel14401-bib-0009] Feng, Y. , Wang, Y. , Wang, P. , Huang, Y. , & Wang, F. (2018). Short‐chain fatty acids manifest Stimulative and protective effects on intestinal barrier function through the inhibition of NLRP3 inflammasome and autophagy. Cellular Physiology and Biochemistry, 49, 190–205. 10.1159/000492853 30138914

[acel14401-bib-0010] Foster, M. A. , Bentley, C. , Hazeldine, J. , Acharjee, A. , Nahman, O. , Shen‐Orr, S. S. , Lord, J. M. , & Duggal, N. A. (2022). Investigating the potential of a prematurely aged immune phenotype in severely injured patients as predictor of risk of sepsis. Immunity & Ageing, 19, 60. 10.1186/s12979-022-00317-5 36471343 PMC9720981

[acel14401-bib-0011] Freund, A. , Laberge, R. M. , Demaria, M. , & Campisi, J. (2012). Lamin B1 loss is a senescence‐associated biomarker. Molecular Biology of the Cell, 23, 2066–2075. 10.1091/mbc.e11-10-0884 22496421 PMC3364172

[acel14401-bib-0012] Ghosh, T. S. , Rampelli, S. , Jeffery, I. B. , Santoro, A. , Neto, M. , Capri, M. , Giampieri, E. , Jennings, A. , Candela, M. , Turroni, S. , Zoetendal, E. G. , Hermes, G. D. A. , Elodie, C. , Meunier, N. , Brugere, C. M. , Pujos‐Guillot, E. , Berendsen, A. M. , De Groot, L. C. P. G. M. , Feskins, E. J. M. , … O'Toole, P. W. (2020). Mediterranean diet intervention alters the gut microbiome in older people reducing frailty and improving health status: The NU‐AGE 1‐year dietary intervention across five European countries. Gut, 69, 1218–1228. 10.1136/gutjnl-2019-319654 32066625 PMC7306987

[acel14401-bib-0013] Gori, A. , Rizzardini, G. , Van't Land, B. , Amor, K. B. , van Schaik, J. , Torti, C. , Quirino, T. , Tincati, C. , Bandera, A. , Knol, J. , Benlhassan‐Chahour, K. , Trabattoni, D. , Bray, D. , Vriesema, A. , Welling, G. , Garssen, J. , & Clerici, M. (2011). Specific prebiotics modulate gut microbiota and immune activation in HAART‐naive HIV‐infected adults: Results of the "COPA" pilot randomized trial. Mucosal Immunology, 4, 554–563. 10.1126/scitranslmed.3006438 21525866 PMC3158294

[acel14401-bib-0014] Groschwitz, K. R. , & Hogan, S. P. (2009). Intestinal barrier function: Molecular regulation and disease pathogenesis. The Journal of Allergy and Clinical Immunology, 124, 3–22. 10.1016/j.jaci.2009.05.038 19560575 PMC4266989

[acel14401-bib-0015] Gui, J. , Zhu, X. , Dohkan, J. , Cheng, L. , Barnes, P. F. , & Su, D. M. (2007). The aged thymus shows normal recruitment of lymphohematopoietic progenitors but has defects in thymic epithelial cells. International Immunology, 19, 1201–1211. 10.1093/intimm/dxm095 17804689

[acel14401-bib-0016] Gulhane, M. , Murray, L. , Lourie, R. , Tong, H. , Sheng, Y. H. , Wang, R. , Kang, A. , Schreiber, V. , Wong, K. Y. , Magor, G. , Denman, S. , Begun, J. , Florin, T. H. , Perkins, A. , Cuív, P. Ó. , McGuckin, M. A. , & Hasnain, S. Z. (2016). High fat diets induce colonic epithelial cell stress and inflammation that is reversed by IL‐22. Scientific Reports, 6, 28990. 10.1038/srep28990 27350069 PMC4924095

[acel14401-bib-0017] Haines, C. J. , Giffon, T. D. , Lu, L. S. , Lu, X. , Lavigne, M. T. , Ross, D. T. , & Lewis, D. B. (2009). Human CD4^+^ T cell recent thymic emigrants are identified by protein tyrosine kinase 7 and have reduced immune function. The Journal of Experimental Medicine, 206, 275–285. 10.1084/jem.20080996 19171767 PMC2646563

[acel14401-bib-0018] Kim, C. O. , Huh, A. J. , Han, S. H. , & Kim, J. M. (2012). Analysis of cellular senescence induced by lipopolysaccharide in pulmonary alveolar epithelial cells. Archives of Gerontology and Geriatrics, 54, e35–e41. 10.1016/j.archger.2011.07.016 21871670

[acel14401-bib-0019] Lee, K. A. , Shin, K. S. , Kim, G. Y. , Song, Y. C. , Bae, E. A. , Kim, I. K. , Koh, C. H. , & Kang, C. Y. (2016). Characterization of age‐associated exhausted CD8^+^ T cells defined by increased expression of Tim‐3 and PD‐1. Aging Cell, 15, 291–300. 10.1111/acel.12435 26750587 PMC4783346

[acel14401-bib-0020] Lim, M. A. , Lee, J. , Park, J. S. , Jhun, J. Y. , Moon, Y. M. , Cho, M. L. , & Kim, H. Y. (2014). Increased Th17 differentiation in aged mice is significantly associated with high IL‐1β level and low IL‐2 expression. Experimental Gerontology, 49, 55–62. 10.1016/j.exger.2013.10.006 24140620

[acel14401-bib-0021] Liu, X. , Mo, W. , Ye, J. , Li, L. , Zhang, Y. , Hsueh, E. C. , Hoft, D. F. , & Peng, G. (2018). Regulatory T cells trigger effector T cell DNA damage and senescence caused by metabolic competition. Nature Communications, 9, 249. 10.1038/s41467-017-02689-5 PMC5770447 29339767

[acel14401-bib-0022] Majumdar, S. , Adiga, V. , Raghavan, A. , Rananaware, S. R. , & Nandi, D. (2019). Comparative analysis of thymic subpopulations during different modes of atrophy identifies the reactive oxygen species scavenger, N‐acetyl cysteine, to increase the survival of thymocytes during infection‐induced and lipopolysaccharide‐induced thymic atrophy. Immunology, 157, 21–36. 10.1111/imm.13043 30659606 PMC6459778

[acel14401-bib-0023] Monaghan, T. M. , Duggal, N. A. , Rosati, E. , Griffin, R. , Hughes, J. , Roach, B. , Yang, D. Y. , Wang, C. , Wong, K. , Saxinger, L. , Pučić‐Baković, M. , Vučković, F. , Klicek, F. , Lauc, G. , Tighe, P. , Mullish, B. H. , Blanco, J. M. , McDonald, J. A. K. , Marchesi, J. R. , … Kao, D. H. (2021). A multi‐factorial observational study on sequential fecal microbiota transplant in patients with medically refractory *Clostridioides difficile* infection. Cells, 10, 3234. 10.3390/cells10113234 34831456 PMC8624539

[acel14401-bib-0024] Nitta, T. , & Takayanagi, H. (2021). Non‐epithelial thymic stromal cells: Unsung heroes in thymus organogenesis and T cell development. Frontiers in Immunology, 11, 620894. 10.3389/fimmu.2020.620894 33519827 PMC7840694

[acel14401-bib-0025] Palmer, D. B. (2013). The effect of age on thymic function. Frontiers in Immunology, 4, 316. 10.3389/fimmu.2013.00316 24109481 PMC3791471

[acel14401-bib-0026] Pangrazzi, L. , Reidla, J. , Carmona Arana, J. A. , Naismith, E. , Miggitsch, C. , Meryk, A. , Keller, M. , Krause, A. A. N. , Melzer, F. L. , Trieb, K. , Schirmer, M. , Grubeck‐Loebenstein, B. , & Weinberger, B. (2020). CD28 and CD57 define four populations with distinct phenotypic properties within human CD8^+^ T cells. European Journal of Immunology, 50, 363–379. 10.1002/eji.201948362 31755098 PMC7079235

[acel14401-bib-0027] Pieren, D. K. J. , Smits, N. A. M. , van de Garde, M. D. B. , & Guichelaar, T. (2019). Response kinetics reveal novel features of ageing in murine T cells. Scientific Reports, 9, 5587. 10.1038/s41598-019-42120-1 30944406 PMC6447543

[acel14401-bib-0028] Salazar, A. M. , Aparicio, R. , Clark, R. I. , Rera, M. , & Walker, D. W. (2023). Intestinal barrier dysfunction: An evolutionarily conserved hallmark of aging. Disease Models & Mechanisms, 16, dmm049969. 10.1242/dmm.049969 37144684 PMC10184675

[acel14401-bib-0029] Saule, P. , Trauet, J. , Dutriez, V. , Lekeux, V. , Dessaint, J. P. , & Labalette, M. (2006). Accumulation of memory T cells from childhood to old age: Central and effector memory cells in CD4(+) versus effector memory and terminally differentiated memory cells in CD8(+) compartment. Mechanisms of Ageing and Development, 127, 274–281. 10.1016/j.mad.2005.11.001 16352331

[acel14401-bib-0031] Seethaler, B. , Lehnert, K. , Yahiaoui‐Doktor, M. , Basrai, M. , Vetter, W. , Kiechle, M. , & Bischoff, S. C. (2023). Omega‐3 polyunsaturated fatty acids improve intestinal barrier integrity‐albeit to a lesser degree than short‐chain fatty acids: An exploratory analysis of the randomized controlled LIBRE trial. European Journal of Nutrition, 62, 2779–2791. 10.1007/s00394-023-03172-2 37318580 PMC10468946

[acel14401-bib-0032] Sempowski, G. D. , Hale, L. P. , Sundy, J. S. , Massey, J. M. , Koup, R. A. , Douek, D. C. , Patel, D. D. , & Haynes, B. F. (2000). Leukemia inhibitory factor, oncostatin M, IL‐6, and stem cell factor mRNA expression in human thymus increases with age and is associated with thymic atrophy. Journal of Immunology, 164, 2180–2187. 10.4049/jimmunol.164.4.2180 10657672

[acel14401-bib-0033] Stehle, J. R., Jr. , Leng, X. , Kitzman, D. W. , Nicklas, B. J. , Kritchevsky, S. B. , & High, K. P. (2012). Lipopolysaccharide‐binding protein, a surrogate marker of microbial translocation, is associated with physical function in healthy older adults. The Journals of Gerontology. Series A, Biological Sciences and Medical Sciences, 67, 1212–1218. 10.1093/gerona/gls178 22960476 PMC3636679

[acel14401-bib-0034] Thevaranjan, N. , Puchta, A. , Schulz, C. , Naidoo, A. , Szamosi, J. C. , Verschoor, C. P. , Loukov, D. , Schenck, L. P. , Jury, J. , Foley, K. P. , Schertzer, J. D. , Larché, M. J. , Davidson, D. J. , Verdú, E. F. , Surette, M. G. , & Bowdish, D. M. E. (2017). Age‐associated microbial dysbiosis promotes intestinal permeability, systemic inflammation, and macrophage dysfunction. Cell Host & Microbe, 21, 455–466. e4. 10.1016/j.chom.2017.03.002 28407483 PMC5392495

[acel14401-bib-0035] Tran, L. , & Greenwood‐Van Meerveld, B. (2013). Age‐associated remodeling of the intestinal epithelial barrier. The Journals of Gerontology. Series A, Biological Sciences and Medical Sciences, 68, 1045–1056. 10.1093/gerona/glt106 23873964 PMC3738030

[acel14401-bib-0036] Tzika, A. A. , Constantinou, C. , Bandyopadhaya, A. , Psychogios, N. , Lee, S. , Mindrinos, M. , Martyn, J. A. , Tompkins, R. G. , & Rahme, L. G. (2013). A small volatile bacterial molecule triggers mitochondrial dysfunction in murine skeletal muscle. PLoS One, 8, e74528. 10.1371/journal.pone.0074528 24098655 PMC3787027

[acel14401-bib-0037] Ueda, K. , Arakawa, H. , & Nakamura, Y. (2003). Dual‐specificity phosphatase 5 (DUSP5) as a direct transcriptional target of tumor suppressor p53. Oncogene, 22, 5586–5591. 10.1038/sj.onc.1206845 12944906

[acel14401-bib-0038] Voetmann, L. M. , Rolin, B. , Kirk, R. K. , Pyke, C. , & Hansen, A. K. (2023). The intestinal permeability marker FITC‐dextran 4kDa should be dosed according to lean body mass in obese mice. Nutrition & Diabetes, 13, 1. 10.1038/s41387-022-00230-2 36604407 PMC9816099

[acel14401-bib-0039] Wei, X. , Zhang, J. , Gu, Q. , Huang, M. , Zhang, W. , Guo, J. , & Zhou, X. (2017). Reciprocal expression of IL‐35 and IL‐10 defines two distinct effector Treg subsets that are required for maintenance of immune tolerance. Cell Reports, 21, 1853–1869. 10.1016/j.celrep.2017.10.090 29141218

[acel14401-bib-0040] Wilson, Q. N. , Wells, M. , Davis, A. T. , Sherrill, C. , Tsilimigras, M. C. B. , Jones, R. B. , Fodor, A. A. , & Kavanagh, K. (2018). Greater microbial translocation and vulnerability to metabolic disease in healthy aged female monkeys. Scientific Reports, 8, 11373. 10.1038/s41598-018-29473-9 30054517 PMC6063974

[acel14401-bib-0041] Yue, S. , Zheng, X. , & Zheng, Y. (2019). Cell‐type‐specific role of lamin‐B1 in thymus development and its inflammation‐driven reduction in thymus aging. Aging Cell, 18, e12952. 10.1111/acel.12952 30968547 PMC6612680

[acel14401-bib-0042] Zhang, Y. H. , Takahashi, K. , Jiang, G. Z. , Kawai, M. , Fukada, M. , & Yokochi, T. (1993). In vivo induction of apoptosis (programmed cell death) in mouse thymus by administration of lipopolysaccharide. Infection and Immunity, 61, 5044–5048. 10.1128/iai.61.12.5044-5048.1993 8225580 PMC281281

[acel14401-bib-0043] Zizzo, G. , De Santis, M. , Bosello, S. L. , Fedele, A. L. , Peluso, G. , Gremese, E. , Tolusso, B. , & Ferraccioli, G. (2011). Synovial fluid‐derived T helper 17 cells correlate with inflammatory activity in arthritis, irrespectively of diagnosis. Clinical Immunology, 138, 107–116. 10.1016/j.clim.2010.10.002 21056009

