# Supplementary material for: Age‐related loss of intestinal barrier integrity plays an integral role in thymic involution and T cell ageing
Source: Aging Cell. 2024 Nov 15;24(3):e14401. doi: 10.1111/acel.14401 (PMC11896561; doi:10.1111/acel.14401)
Supplement: Supplementary file 1 — Data S1: [file ACEL-24-e14401-s001.pdf]

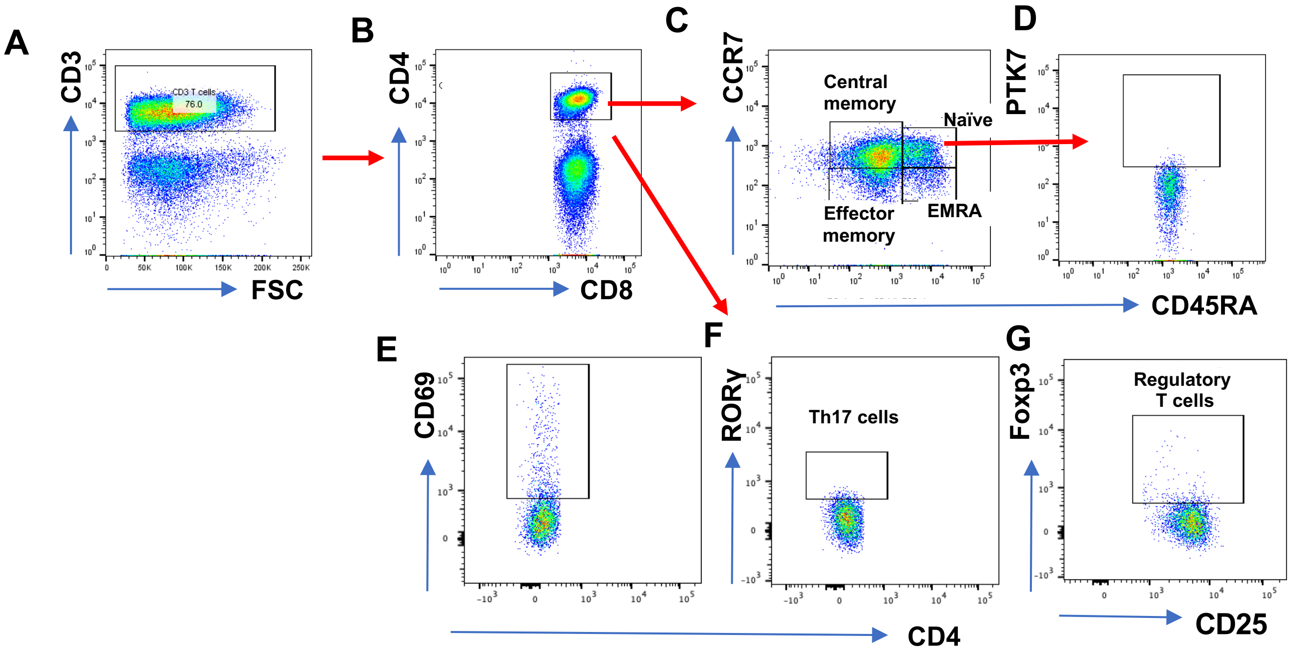

Supplementary Figure 1: T cell subset gating strategy.

		Occludin (ng/ml)		LBP (ng/ml)		sCD14 (pg/ml)	
		R	p	R	p	R	p
Young adults (n = 27)	BMI (kg/m²)	-0.02	0.92	**-0.4**	**0.05**	-0.12	0.59
	Physical activity levels (MET)	0.26	0.19	0.34	0.08	0.15	0.46
	Sedentary TV viewing time (hrs)	-0.29	0.14	-0.35	0.08	**0.52**	**0.006**
	Sleep quality (PSQI)	-0.14	0.49	-0.11	0.57	-0.01	0.96
	HADS anxiety score	-0.08	0.68	-0.11	0.57	-0.1	0.62
	HADS depression score	-0.1	0.63	-0.12	0.56	-0.1	0.62
	Mediterranean Diet Score	0.05	0.8	0.06	0.78	0.02	0.92
	Diet Quality Index	-0.17	0.39	0.06	0.78	-0.22	0.27
Old adults (n = 55)	BMI (kg/m²)	-0.08	0.6	-0.14	0.4	-0.04	0.79
	Physical activity levels (MET)	-0.01	0.94	0.07	0.65	0.08	0.63
	Sedentary TV viewing time (hrs)	-0.03	0.86	-0.19	0.24	-0.15	0.36
	Sleep quality (PSQI)	0.08	0.62	-0.2	0.21	-0.13	0.44
	HADS anxiety score	-0.25	0.13	0.14	0.4	-0.14	0.39
	HADS depression score	0.2	0.21	-0.14	0.4	-0.3	0.06
	Mediterranean Diet Score	-0.3	0.06	0.24	0.14	0.25	0.13
	Diet Quality Index	-0.07	0.68	-0.08	0.64	0.05	0.76

Supplementary Table 1: Links between microbial translocation and lifestyle factors.

	Low MT young (n = 26)	Low MT old (n = 13)	High MT old (n = 25)	P-value
Actinobacteria				
Adlercreutzia	3.4 ± 2.2	0.8 ± 0.3	2.6 ± 1.3	0.51
Bifidobacterium	86.9 ± 3.5	57 ± 7.4	66.4 ± 5.7	**0.0002**
Collinsella	4.9 ± 1.5	7.8 ± 1.5	8.8 ± 2.2	0.28
Eggerthella	0.7 ± 0.4	0.1 ± 0.1	0.3 ± 0.2	0.23
Bacteroidetes				
Allistipes	6.6 ± 1.4	9.3 ± 4.5	10.9 ± 2.3	0.1
Bacteroides	65.3 ± 6.2	51.4 ± 10.1	52.5 ± 5.8	0.18
Barnesiella	1.5 ± 0.4	1 ± 0.4	4.2 ± 2.1	0.32
Paraprevotella	1.8 ± 1.3	0.9 ± 0.8	0.8 ± 0.5	0.44
Prevotella	0.01 ± 0.01	0.05 ± 0.04	0.001 ± 0.001	0.29
Cyanobacteria				
Gastranaerophilales	30.8 ± 9.2	61.5 ± 14	60 ± 10	0.07
Firmicutes				
Anaerotruncus	0.02 ± 0.01	0.01 ± 0.01	0.02 ± 0.004	0.82
Blautia	11 ± 1.2	8.9 ± 2.1	6.1 ± 1.3	**0.002**
Christensenellaceae R-7 group	1.4 ± 0.5	3.2 ± 1.4	6.6 ± 1.6	**0.02**
Clostridia	0.1 ± 0.1	0.03 ± 0.01	0.05 ± 0.02	0.34
Coprococcus	2.2 ± 0.6	2.3 ± 0.8	1.8 ± 0.5	0.9
Dialister	1.6 ± 0.4	2.1 ± 0.6	1.1 ± 0.3	0.23
Faecalibacterium	22.1 ± 2.6	21.6 ± 4.5	13.4 ± 1.9	0.06
Lachnospiraceae	3.7 ± 0.3	3 ± 0.6	2.8 ± 0.6	**0.01**
Lactobacillus	0.02 ± 0.01	0.02 ± 0.01	0.05 ± 0.04	0.07
Oscillibacter	0.1 ± 0.02	0.06 ± 0.02	0.1 ± 0.03	0.32
Peptostreptococcaceae	0.001 ± 0.0005	0 ± 0	0 ± 0	0.1
Roseburia	2.6 ± 1.3	1.5 ± 0.7	1.8 ± 0.4	0.19
Ruminiclostridium	0.2 ± 0.1	0.2 ± 0.1	0.5 ± 0.2	0.07
Ruminococcaceae	0.6 ± 0.1	0.9 ± 0.2	1.3 ± 0.2	**0.03**
Fusobacteria				
Fusobacterium	3.8 ± 3.8	23.1 ± 12.2	5.3 ± 4.2	0.14
Proteobacteria				
Bilophila	15.3 ± 3.4	7.5 ± 3.1	12.3 ± 3.4	0.29
Desulfovibrio	11.9 ± 4	8.3 ± 5	7 ± 3.2	0.82
Escherichia-Shigella	13.2 ± 4.4	22.7 ± 10.3	16.1 ± 5.5	0.74
Sutterella	40.1 ± 6.7	14.6 ± 5	19.7 ± 4	**0.02**
Tenericutes				
Anaeroplasma	15.1 ± 7	6 ± 6	9.6 ± 5	0.81
Verrucomicrobia				
Akkermansia	96.2 ± 3.8	100 ± 0.01	100 ± 0	0.43

Supplementary Table2: Faecal microbiota composition of young and old adults displaying low and high microbial translocation.

	Low MT young (n = 27)	Low MT old (n = 24)	High MT old (n = 31)	P-value
**CD4 T cells (%)**	38.9 ± 2	41.6 ± 3.3	47.6 ± 2.2	**0.02**
**CD4 T cells (10^9/L)**	0.5 ± 0.04	0.4 ± 0.1	0.5 ± 0.04	0.57
**Naïve CD4 T cells (%)**	50.9 ± 2.5	11.7 ± 2.4	18.8 ± 2.6	**<0.0001**
**Naïve CD4 T cells (10^9/L)**	0.3 ± 0.03	0.05 ± 0.01	0.1 ± 0.03	**<0.0001**
**Central memory CD4 T cells (%)**	16.1 ± 1.4	14.5 ± 2.9	12.3 ± 1.4	0.1
**Central memory CD4 T cells (10^9/L)**	0.08 ± 0.01	0.05 ± 0.01	0.06 ± 0.01	**<0.0001**
**Effector memory CD4 T cells (%)**	27.9 ± 1.6	49.2 ± 3.1	35.7 ± 2.4	**<0.0001**
**Effector memory CD4 T cells (10^9/L)**	0.1 ± 0.01	0.2 ± 0.03	0.2 ± 0.02	**0.01**
**EMRA CD4 T cells (%)**	8.7 ± 1	22.8 ± 3	29.5 ± 3.1	**<0.0001**
**EMRA CD4 T cells (10^9/L)**	0.04 ± 0.002	0.1 ± 0.02	0.1 ± 0.02	**<0.0001**
**Total memory CD4 T cells (%)**	52.7 ± 2.5	86.5 ± 2.4	80.8 ± 2.4	**<0.0001**
**Total memory CD4 T cells (10^9/L)**	0.2 ± 0.02	0.4 ± 0.05	0.4 ± 0.04	**0.001**
**CD69^{+ve} CD4 T cells (%)**	3.5 ± 0.4	5.7 ± 1.1	5.5 ± 0.4	**0.003**
**CD154^{+ve} CD4 T cells (%)**	28.3 ± 2.6	29.8 ± 2.5	31.6 ± 2.2	0.6
**CD28^{-ve}CD57^{+ve} CD4 T cells (%)**	12.3 ± 1.8	16.8 ± 4	27.2 ± 4.4	**0.03**
**PD1^{+ve} CD4 T cells (%)**	28.9 ± 2.6	32 ± 4.5	31.7 ± 2.3	0.46
**Cytokine production**				
**IL4^{+ve} CD4 T cells (%)**	6.1 ± 0.7	7.7 ± 1.7	7.7 ± 0.92	0.41
**IL4 expression in CD4 T cells (MFI)**	736 ± 65.2	650 ± 64.9	754.8 ± 65.4	0.59
**IFNγ^{+ve} CD4 T cells (%)**	15.8 ± 1.6	22.1 ± 2.4	18.8 ± 2	0.11
**IFNγ expression in CD4 T cells (MFI)**	1515 ± 141.1	1479 ± 178.2	1504 ± 128.4	1

Supplementary Table 3: CD4 T cell subset distribution.

	Low MT young (n = 27)	Low MT old (n = 24)	High MT old (n = 31)	P-value
**CD8 T cells (%)**	44.1 ± 1.6	41.9 ± 2.7	37.4 ± 1.8	**0.03**
**CD8 T cells (10^9/L)**	0.5 ± 0.04	0.4 ± 0.05	0.4 ± 0.03	**0.03**
**Naïve CD8 T cells (%)**	41.7 ± 2.5	11.8 ± 1.6	10 ± 1.1	**<0.0001**
**Naïve CD8 T cells (10^9/L)**	0.2 ± 0.02	0.04 ± 0.01	0.05 ± 0.01	**<0.0001**
**Central memory CD8 T cells (%)**	4.8 ± 0.6	8.8 ± 1.9	10.9 ± 1.8	**0.03**
**Central memory CD8 T cells (10^9/L)**	0.02 ± 0.01	0.03 ± 0.01	0.04 ± 0.01	0.44
**Effector memory CD8 T cells (%)**	12.2 ± 1	27.5 ± 2.6	19.6 ± 2.6	**<0.0001**
**Effector memory CD8 T cells (10^9/L)**	0.07 ± 0.01	0.09 ± 0.01	0.06 ± 0.01	0.07
**EMRA CD8 T cells (%)**	42 ± 2.3	51.7 ± 3.8	57.9 ± 3.3	**0.001**
**EMRA CD8 T cells (10^9/L)**	0.2 ± 0.02	0.2 ± 0.03	0.2 ± 0.02	0.87
**Total memory CD8 T cells (%)**	61.4 ± 2.6	86 ± 1.8	89.1 ± 1.1	**<0.0001**
**Total memory CD8 T cells (10^9/L)**	0.3 ± 0.03	0.4 ± 0.05	0.3 ± 0.04	0.45
**CD69^{+ve} CD8 T cells (%)**	5.8 ± 0.4	9.7 ± 0.9	12.5 ± 1.1	**<0.0001**
**CD154^{+ve} CD8 T cells (%)**	21 ± 1.8	24.7 ± 3	26.7 ± 1.8	0.1
**Cytokine production**				
**IFNγ^{+ve} CD8 T cells (%)**	12.3 ± 1.6	14.4 ± 2	15.1 ± 1.6	0.38
**IFNγ expression in CD8 T cells (MFI)**	1175 ± 110.2	1171 ± 174	1229 ± 111.3	0.74
**TNFα^{+ve} CD8 T cells (%)**	3.7 ± 0.4	3.7 ± 0.5	3.4 ± 0.4	0.75
**TNFα expression in CD8 T cells (MFI)**	441.1 ± 79.2	333 ± 56.8	424.1 ± 62.9	0.83

Supplementary Table 4: CD8 T cell subset distribution.

	**Low MT young** (n = 15)	**Low MT old** (n = 14)	**High MT old** (n = 20)	**P-value**
**IL1ß (pg/ml)**	7.9 ± 2.7	6 ± 3	7.6 ± 1.8	0.88
**IL4 (pg/ml)**	109.2 ± 25.6	72.3 ± 22.7	132.1 ± 33.1	0.31
**IL6 (pg/ml)**	3.6 ± 1	3.1 ± 0.7	2.2 ± 0.6	0.42
**IL7 (pg/ml)**	7.4 ± 1.1	9.4 ± 1.7	7.8 ± 0.8	0.49
**IL10 (pg/ml)**	8.6 ± 3.3	4.8 ± 2.4	7.3 ± 1.9	0.49
**IL15 (pg/ml)**	6.6 ± 1.4	7 ± 1.5	8.5 ± 2.5	0.85
**IL17 (pg/ml)**	4.7 ± 3.9	4.3 ± 1.8	2.9 ± 1.2	0.51
**TNFα (pg/ml)**	3.8 ± 0.7	6.9 ± 1.9	4.1 ± 0.5	0.56
**CRP (µg/ml)**	2.7 ± 0.7	3.1 ± 1.2	1.9 ± 0.3	1
**IFNγ (pg/ml)**	145.7 ± 48.4	186.6 ± 86	297.8 ± 123.5	0.63
**CXCL9 (pg/ml)**	3958 ± 897.3	2696 ± 788.6	2801 ± 656.7	0.46
**GM-CSF (pg/ml)**	34.2 ± 7.9	80.5 ± 25	54.6 ± 18.8	0.18

Supplementary Table 5: Circulating inflammatory markers.

	Low MT young mean gene expression	Low MT old mean gene expression	Fold change	P-value	Gene function
Downregulated					
**ALCAM**	60.2	33.8	-1.43	**0.05**	Leukocyte adhesion molecule [71]
**BID**	46.9	26.1	-1.52	**0.04**	Pro-apoptotic molecule [72]
**BLNK**	57.4	27.8	-1.69	**0.03**	BCR signaling [73]
**CR2**	41.3	21.7	-2.04	**0.01**	Complement receptor that clears opsonized immune complexes [28]
**LRRN3**	71.3	20.1	-3.02	**0.05**	Biomarker for Parkinson's disease and suppressor of immune responses [74]
**NT5E**	41.1	23.5	-1.74	**0.04**	Immune inhibitory molecule and regulator of cellular signaling [75,76]
**TNFRSF13B**	49.8	20.1	-2.22	**0.05**	Controls immune-mediated pathology and enhances defense against pathogens [77]

Supplementary Table 6: Genes differentially expressed in peripheral blood mononuclear cells (PBMCs) from low MT young and low MT old adults.

	Low MT young mean gene expression	High MT old mean gene expression	Fold change	P-value	Gene function
Downregulated					
**ALCAM**	60.2	40.4	-1.39	0.05	Leukocyte adhesion molecule [71]
**AMICA1**	1089.2	724.8	-1.4	0.04	Leukocyte transmigration [78]
**ATG7**	228.1	154.5	-1.52	0.05	Autophagy regulator [79]
**ATM**	27.1	19.2	-1.28	0.05	DNA damage repair [80]
**CCR7**	211.7	184.4	-1.24	0.05	Chemokine receptor facilitating recruitment and retention of immune cells in lymphoid organs [81]
**CD28**	108.1	79.4	-1.39	0.05	Costimulatory receptor involved in T cell activation whose loss is a marker for cellular senescence [82]
**CLEC6A**	33.4	21.5	-1.63	0.03	Pattern recognition receptor [83]
**CR1**	647.3	367	-1.64	0.05	Complement receptor that clears opsonized immune complexes [28]
**EGR1**	90.8	35.2	-2.36	0.04	Transcriptional regulator [84]
**FOS**	6612.7	2861.6	-2.15	0.04	Regulator of proliferation and senescence onset [85,86]
**IRF5**	89.6	64.9	-1.28	0.05	Interferon regulatory transcription factor [87]
**LAMP1**	959.4	841	-1.02	0.03	Degradation of autophagic and lysosomal organelles [88]
**MAPK3**	243.8	199.9	-1.13	0.04	Regulates proliferation, differentiation and cell cycle arrest [29]
**MEFV**	164.7	96.7	-1.58	0.04	Inflammasome complex assembly and regulates inflammation [89]
**PTGS2**	162.9	32.6	-4.97	0.02	Controls prostaglandin production [90]
**TLR4**	313.4	195.8	-1.49	0.05	Pattern recognition receptor [91]
**TLR8**	119.5	87.5	-1.27	0.05	Pattern recognition receptor [92]
Upregulated					
**BCL2**	278.7	331.3	1.24	0.05	Anti-apoptotic molecule [93]
**CASP3**	84.1	139.1	1.52	0.05	Pro-apoptotic molecule [94]
**CD99**	1473.7	1836.2	1.34	0.05	Immature T cell marker, regulator of cytokine production and apoptosis inducer [95,96]
**DUSP4**	26.3	52.4	1.73	0.04	Regulates MAPKs and cellular proliferation and accelerates T cell senescence [97,98]
**DUSP6**	598	1456.3	1.8	0.04	Regulates MAPKs and cellular proliferation [97]
**ICOS**	91.1	132	1.44	0.05	Costimulatory molecule essential for T cell activation and proliferation [99]
**KLRG1**	214.9	360.8	1.23	0.04	Co-inhibitory receptor and cellular senescence marker [100]
**NEFL**	22.4	34.7	1.82	0.04	Blood biomarker of axonal damage [101]
**RORA**	420	588.4	1.51	0.03	Th17 polarization [102]
**TXNIP**	11200.8	14076.1	1.35	0.05	Cell cycle arrest inducer [103]

Supplementary Table 7: Genes differentially expressed in peripheral blood mononuclear cells (PBMCs) from low MT young and high MT old adults.

	Low MT old mean gene expression	High MT old mean gene expression	Fold change	P-value	Gene function
Downregulated					
ATG7	178.5	154.5	-1.25	0.01	Autophagy regulator [79]
BID	26.1	23.9	-1.27	0.04	Pro-apoptotic molecule [72]
CD99	1444.4	1836.2	-1.09	0.01	Immature T cell marker, regulator of cytokine production and apoptosis inducer [95,96]
CLEC6A	55.2	21.5	-1.39	0.003	Pattern recognition receptor [83]
CR1	613.4	367	-2.09	0.04	Complement receptor that clears opsonized immune complexes [28]
G6PD	550.7	466.8	-1.04	0.03	Glycolytic enzyme that protects against DNA damage repair and contributes to T cell ageing [104,105]
HAVCR2	436.8	290.5	2.8	0.05	Cellular exhaustion inducer and negative regulator of tolerance [106]
HLA-DRB4	62.3	2290.3	-1.07	0.05	Antigen presenting molecule [107]
IL12RB1	171.8	151.5	-1.64	0.01	Receptor that binds to pro-inflammatory cytokines IL12 and IL23 [108]
IL12RB2	33.3	31.2	-1.5	0.05	Receptor that binds to pro-inflammatory cytokines IL12 and IL23 [108]
IRF5	95.9	64.9	-1.04	0.05	Interferon regulatory transcription factor [87]
LILRB2	1218.5	716.5	-3.6	0.04	Signal transduction via binding to MHC class I molecules on antigen-presenting cells [109]
TLR4	325.6	195.8	-1.31	0.04	Pattern recognition receptor [91]
Upregulated					
CASP8	940.8	1262.9	1.7	0.04	Pro-apoptotic molecule [110]
DUSP4	30.9	52.4	1.08	0.04	Regulates MAPKs and cellular proliferation and accelerates T cell senescence [97,98]
DUSP6	1031.4	1456.3	1	0.05	Regulates MAPKs and cellular proliferation [97]
HMGB1	423.3	521.5	1.06	0.01	DAMP that activates pro-inflammatory signalling pathways [111]
IL18R1	108.7	174.4	1.02	0.04	Receptor that binds to the pro-inflammatory cytokine IL18 [112]
MFGE8	70.3	102.3	1.31	0.05	Biomarker of arterial ageing [113]
POU2AF1	23.1	45.7	1.02	0.05	B cell transcriptional coactivator [114]
ZAP70	1168.4	1397.6	1.02	0.05	TCR signaling [115]

Supplementary Table 8: Genes differentially expressed in PBMCs from low MT old and high MT old adults.
